# Scanning and Actuation Techniques for Cantilever-Based Fiber Optic Endoscopic Scanners—A Review

**DOI:** 10.3390/s21010251

**Published:** 2021-01-02

**Authors:** Mandeep Kaur, Pierre M. Lane, Carlo Menon

**Affiliations:** 1 MENRVA Research Group, Schools of Mechatronic Systems Engineering and Engineering Science, Simon Fraser University, Surrey, B.C. V3T 0A3, Canada; mka116@sfu.ca; 2 School of Engineering Science, Simon Fraser University, Burnaby, B.C. V5A 1S6, Canada; plane@bccrc.ca; 3 Imaging Unit, Integrative Oncology, BC Cancer Research Center, Vancouver, B.C., V5Z 1L3, Canada

**Keywords:** endoscopes, medical imaging, MEMS actuators, piezoelectric, electrothermal, electrostatic, electromagnetic, shape memory alloys, scanning patterns

## Abstract

Endoscopes are used routinely in modern medicine for in-vivo imaging of luminal organs. Technical advances in the micro-electro-mechanical system (MEMS) and optical fields have enabled the further miniaturization of endoscopes, resulting in the ability to image previously inaccessible small-caliber luminal organs, enabling the early detection of lesions and other abnormalities in these tissues. The development of scanning fiber endoscopes supports the fabrication of small cantilever-based imaging devices without compromising the image resolution. The size of an endoscope is highly dependent on the actuation and scanning method used to illuminate the target image area. Different actuation methods used in the design of small-sized cantilever-based endoscopes are reviewed in this paper along with their working principles, advantages and disadvantages, generated scanning patterns, and applications.

## 1. Introduction

An endoscope is an imaging device made up of a long and thin tube that can be inserted into the hollow openings of the body to image the inner sections in real time and in a less invasive manner. Advances in fiber optic systems led to the development of flexible endoscopes, enabling high-resolution images of narrow sections of the body and reducing the number of biopsies required for a specific diagnosis, with applications such as cancer detection, microvascular oxygen tension measurement, chronic mesenteric ischemia, subcellular molecular interactions, etc. Earlier developed standard white light endoscopes (WLEs) had limited ability to differentiate metaplasia from dysplasia. Such limitations were surpassed by enhancing the image contrast through the use of dyes in chromoendoscopy or applying digital filters in narrow band imaging (NBI) [1,2]. The increased use of endoscopic devices highly improved the diagnostic rate of cancers by permitting the visualization of early dysplasias which may lead to cancer development [1]. Cancer is the second leading cause of death in the world. Approximately one out of every six deaths are due to cancer, killing 9.6 million individuals worldwide in 2018 [3]. It has been observed that early detection has significantly improved life expectancy and reduced the mortality rate by 30–40% over the last two decades [4].

One of the recently advanced imaging devices was based on confocal laser endoscopy (CLE). Two variations of CLE that are used commercially in medical applications are the so-called e-CLE, which is an integrated confocal endoscope developed by Pentax Medical (Tokyo, Japan), and the probe-based confocal microscope (p-CLE) developed by Mauna Kea Technologies (Paris, France) [5]. Optical coherence tomography (OCT) is another frequently used technique to image tissue, where a change in the refractive index of the scattering coefficient alters the intensity of the backscattered light and is used to provide contrast in the image [6]. Photoacoustic (PA) imaging is another imaging technology that images a tissue surface using short pulsed light waves and detects the ultrasonic waves generated from the optical absorption. It integrates the benefits of high contrast in optical imaging and deep penetration of the ultrasonic imaging [7]. On the basis of imaging depth, PA imaging can be classified into PA microscopy (penetration depth < 10 mm), PA computed tomography (penetration depth between 10 and 100 mm), and minimally invasive PA imaging (penetration depth ≥ 100 mm) [8]. In addition to these techniques, there is visible light spectroscopy (VLS), where the tissue surface is exposed to visible light and the absorbance spectrum provides structural and functional information about the tissue. This technique is largely used to monitor the microvascular hemoglobin oxygen saturation, which can be further used to evaluate local ischemia situations by measuring the difference between oxygenated and deoxygenated hemoglobin [9,10].

The advancement in optics along with the development of micro-electro-mechanical systems (MEMS) and microfabrication techniques led to the fabrication of sub-millimeter-sized flexible endoscopes that can image the narrow cavities in the body, providing information about early-stage pre-cancerous tissues. The optimized final design of an endoscope is often the result of an optical design optimized using special software-based computer simulations [11]. The rapid evolvement of the software and fabrication technologies enabled endoscopes to capture the tissue images in three-dimensional space, providing in depth information about the target surface as well [12]. Most of the preliminary video endoscopes used coherent optical fiber bundles (CFBs) to transport light from a light source, such as a xenon lamp or a laser light, to the imaged surface and used charge-coupled devices (CCDs) to image the tissue surface [13]. Those CCD devices contained approximately 200,000 pixels, which provided limited resolution of the image [2]. The image resolution and optical magnification in newly developed endoscopes was enhanced using complementary metal oxide semiconductor (CMOS) chips, which provided images with over 1.3 MPixels of diffraction-limited resolution [14]. However, a minimum center-to-center distance between the optical fibers in a CFB and the honeycomb effect produced by the non-imaging area between fibers still limited the resolution in devices having a diameter smaller than 3 mm, independently of the imaging chip used [15].

It is possible to obtain high-spatial-resolution images with flexible endoscopes having sub-millimeter diameters by scanning the laser light at the proximal end and capturing the image on a temporal basis, i.e., acquiring one pixel at a time. Seibel et al. from the University of Washington developed such a cantilever-based scanning fiber endoscope (SFE), where a single-mode fiber was excited at resonance to scan the light beam on the target area, and an outer ring of optical fibers captured the backscattered light [16]. Since that development, a large number of cantilever-based imaging devices have been fabricated, which will be discussed later in the paper. In such devices, the tip displacement of an optical fiber acting as a cantilever beam dictates the field of view (FOV) and the resolution of the obtained image. Scanning and actuation techniques used to excite a cantilever beam play a critical role in the performance of such a scanning device. In addition, the small size and the distortion-free imaging requirements need to be considered during the design of an endoscopic device for medical purposes as the size of an imaging probe sets the targeted imageable area, and the motion of organs can lead to the generation of artifacts in the image.

A large number of state-of-the-art reviews on endoscopic imaging devices are available in the literature which are mainly focused on different imaging modalities. The review work in [17] provided a general description of confocal microscopy, OCT, and two-photon imaging modalities. Similarly, the review work in [18] investigated various imaging modalities with some information on their actuation mechanisms. The previous work [6] and the review article by Hwang et al. in [19] provided a general description of various endoscopic imaging technologies, modalities, packaging/scanning configurations, and actuation mechanisms. However, a detailed review of the different actuation mechanisms used in fiber optic endoscopic scanners has not been provided previously. For this reason, the scanning and actuation techniques used in the recently developed cantilever-based fiber optic scanners for medical purposes are described in this paper in detail. In the current review, the mathematical models and applications of MEMS actuators in fiber optic cantilever-based scanners are reported to provide information about the underlying working physics of these devices and can provide a foundation for the development of miniaturized and more efficient MEMS scanners.

The paper is organized as follows: Section 2 provides an overview of the optical components used in endoscopic devices. Scanning techniques used in such small-sized devices are described in Section 3. Section 4 describes the various actuation methods used to excite the miniaturized optical cantilever beams in detail. A discussion and conclusions about available cantilever-based scanning devices are reported in Section 5 and Section 6, respectively.

## 2. Overview of Optical Components Used in Endoscopes

The key element used in cantilever-based endoscopic devices is an optical fiber acting as a waveguide through which light is transmitted using the principle of total internal reflection at the interface of two different dielectric media. The inner cylindrical portion is called the core, while the outer region is named the cladding. Both materials are characterized by a slight change in refractive index; it is from this that the name of step-index fibers comes. There are different classifications of optical fibers available based on normalized wave number *V*, defined as:

(1) V=(2πaNA)/λ

where a is the core radius, *λ* is the wavelength of the light, and *NA* is the numerical aperture of the fiber [20]. *NA* is calculated from the refractive index of the core and cladding material of the fiber as [20]:

(2) $$NA=\sqrt{n^{2}{}_{core}-n^{2}{}_{clad}}$$

The *V*-parameter determines the number of spatial modes of the electromagnetic wave that can propagate within the fiber. An optical fiber is defined as a single mode fiber (SMF) if *V* < 2.405.

For a large *V*-parameter, the total number of allowed modes *N* through the fiber can be approximated as [20]:

(3) $$N\approx V^{2}/2$$

Such fibers are called multimode fibers (MMF).

Other than simple step-index fibers, there are other fibers having different refractive index profiles with radius and they are increasingly finding use in communication fields to avoid the dispersion of optical waves during propagation. Among these fibers, the most commonly used ones are fibers having a double-step-shaped profile or a nearly parabolic variation of index designated double-clad fibers (DCF) and gradient-index (GRIN) fibers, respectively. In GRIN fibers, light propagates due to the profile of the refractive index instead of the total internal reflection and follows a sinusoidal path [20].

Different kinds of optical imaging modalities used in endoscopic imaging devices are described in detail in this section. These technologies are compared with a CCD/CMOS camera in Table 1. Following the definitions in [15], pixel density for the CFB, CCD, and CMOS technologies is the number of imaging elements (pixels or fibers) per square millimeter that can be achieved in practice, while image resolution is the number of these elements in a 1mm diameter. Pixel density and image resolution for SFE assume a single-mode fiber and are based on specific sampling considerations [15].

### 2.1. Fiber Bundles in Endoscopes

Fiber bundles comprise thousands of step-index (or GRIN) fibers contained within a very small area, ranging from a few micrometers to millimeters in range. A fiber bundle is used to carry the illumination light from the proximal end to the distal end, and vice versa, of a device. In a coherent fiber bundle, fibers at both ends are placed at the same relative position, so that the image is transmitted from one end to the other without any distortion. In miniaturized scanners, a coherent fiber bundle is predominantly used due to the perfect alignment of fibers, which facilitates the decoding of the signal [21]. As the light intensity information is transmitted from one end of the fiber bundle to the other, it is possible to place the scanning mechanism at the proximal end of the device, which is a major advantage of such devices.

Every core of the step-index fiber in the bundle acts as a pixel of the imaged data. Thus, the resolution of the image captured using a fiber bundle depends on the core size of the fiber and the core-to-core distance between the fibers [22]. For a coherent fiber bundle with the core separation distance among adjacent fibers represented by Δ*_core_*, the cross-sectional core density or pixel density is given by [15]:

(4) $$Core\;density=\frac{2}{\sqrt{3}}{\left(\frac{1}{\Delta_{core}}\right)}^{2}$$

From the core density and the active area covered by the fibers *A_cfb_*, neglecting the space occupied by outer protective jacket and sheath, the image resolution can be obtained as [15]:

(5) $$Image\;resolution=core\;density*A_{cfb}$$

This equation indicates a major drawback of using fiber bundles for miniaturized devices as the resolution will be poor due to the honeycomb effect generated by the space between the consecutive fiber cores representing the non-imaged area [15].

Another disadvantage of using fiber bundles is the crosstalk phenomenon. The cladding of the step-index fibers constituting the bundle is rendered to a thin layer around the core during the fabrication of a flexible fiber bundle. A very thin cladding surface enables the leakage of the light as it travels through the core, reducing the contrast and the resolution of the image generated. It is possible to reduce the effect of crosstalk by increasing the thickness of the cladding section, enlarging contrast between the refractive indices of the core and cladding material, and/or by reducing the number of modes propagating through the fiber. However, these approaches make the device rigid, and the honeycomb effect will be more intensified, thus degrading the resolution [23,24].

It is possible to refine the resolution of the obtained images by post-processing the data using certain algorithms. Using specific transformation models based on the point spread function of the cores of each fiber from the bundle, it is possible to smooth the light gradient, reducing the pixilation effect given by the honeycomb pattern [25,26,27]. The limitation of these transformation methods to be able to improve the resolution due to under-sampling is alleviated through the use of pixel super-resolution techniques. In such methods, the source/image probe is shifted slightly a number of times for the acquisition of different images, and these images are further combined to enhance the resolution of the image [28].

**Figure 1:**
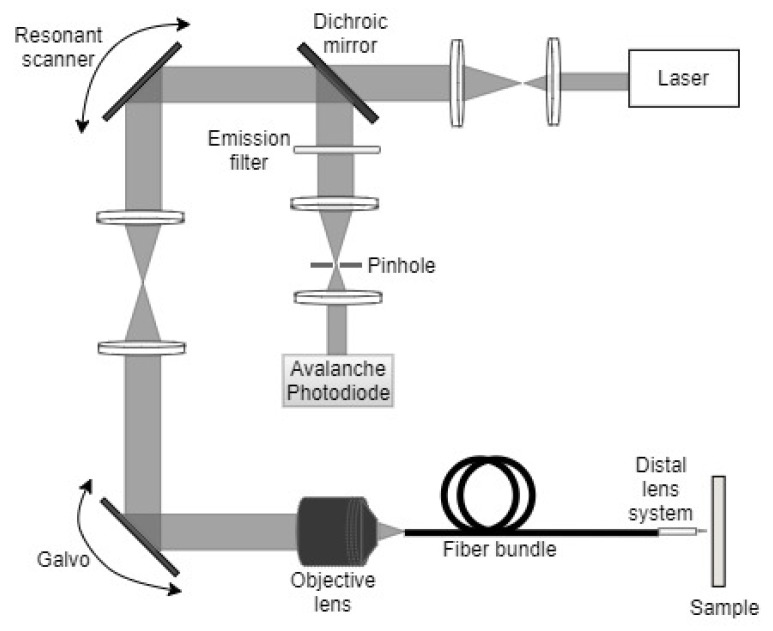
Block diagram of a confocal micro-endoscope.

In endoscopes that use fiber bundles, a pair of mirrors placed at the proximal end of the device permit the illumination of a single fiber from the bundle at a time. The fast-axis scanning (axis with high scanning frequency) is performed using the resonant scanner (mirror surface vibrated at resonance using one of the actuators described later in the paper), while a galvo scanner (optical mirror scanned using a galvanometer-based motor) is used for the slow-axis scanning. A schematic diagram of a confocal micro-endoscope developed using such a technique is shown in Figure 1.

Sung et al. developed an early fiber-optic-based confocal reflectance microscope to image epithelial cells and tissues in real time, showing the cell morphology and tissue architecture without the use of any fluorescent stains. The image guide contained 30,000 fibers and the device had an overall diameter of 7 mm and rigid length of 22 mm to image human tissue with a lateral and axial resolution (smallest resolvable feature) of 2 µm and 10 µm, respectively [29]. Knittel et al. developed a similar confocal endoscope where tissue images with a lateral and axial resolution of 3.1 µm and 16.6 µm, respectively, were obtained using a similar fiber bundle contained in a diameter of 1 mm [30]. Lane et al. developed a similar endoscopic probe for imaging bronchial epithelium having a diameter and length of 1.27 mm and 10 mm, respectively. This probe had a lateral resolution of 1.4 µm and an axial resolution of 16 µm–26 µm [31]. A commercially available endoscope based on this technique was developed by Mauna Kea Technologies [32,33].

### 2.2. Single Fiber Endoscopy

A single-mode fiber (SMF), characterized by having a step-shaped index of refraction change from the core of the fiber to the cladding surface, permits the propagation of only one mode of light through the fiber. A single spatial mode enables a diffraction-limited spot to be projected on the sample plane, resulting in a high-resolution image with the use of an SMF. Due to this property and high flexibility, such fibers find use in miniaturized optical scanners.

An SMF may find a use in a scanning fiber optic microscope, acting as a spatial filter [34] or as a pinhole detector [35]. The same fiber is used for laser light illumination and the collection of the reflected light [34]. An SMF serves as a pinhole in a confocal system and is used in spectrally encoded confocal microscopes [36,37]. By moving the fiber in a plane perpendicular to its axis using mechanical systems or a galvanometer, it is possible to obtain a 2D image.

The SFE described earlier uses an SMF vibrated in resonance to scan the light beam across the target tissue surface, and a peripheric ring of fibers detects the time-multiplexed backscattered light. In this case, the sample resolution depends on the scanning motion and sampling rate, which are not fixed a priori during fabrication. In an SFE, the smallest resolvable feature is determined by its point spread function. A wider tip displacement will provide a higher FOV and higher image resolution in terms of the number of pixels in the scanned area, as described in Table 1.

In contrast to an SMF, a multimode fiber can transmit a large number of spatial modes at the same time. These fibers have core sizes much larger than the single-mode fibers, usually in the range of 50 µm–2000 µm. Multimode fibers can be classified into step-index multimode fibers and graded-index (GRIN) multimode fibers on the basis of the change in refractive index from the core to the cladding, which can be sharp or gradual, respectively [38].

A multimode fiber can be considered as an alternative to a fiber bundle and supports the miniaturization of optical devices. As each fiber in the fiber bundle represents a pixel for the acquired image, each pixel can be represented by a propagating mode in the fiber. Thus, it is possible to increase the pixel density of a device by up to 1–2 orders of magnitude by replacing the fiber bundle with a multimode fiber [39]. A side-viewing endoscopic probe for PA and ultrasound (US) imaging is developed using an MMF to deliver laser pulses to the target tissue, and a coaxial US transducer detects the PA and US echo signals. The light and acoustic signal is deflected 45° by a scanning mirror placed at the distal end of the probe, which is rotated by magnets or a micromotor to provide a rotational scanning [40,41].

The main limitation of using a multimode fiber in an imaging device is modal dispersion, which causes multipath artifacts. Several methods have been explored to provide an image without image artifacts. For example, Papadopoulos et al. used a digital phase conjugation technique to generate a sharp focus point. In this technique, the phase of the distorted wavefront was calculated and an unmodulated beam of this phase was propagated in a backward direction to cancel out the distortions and to generate the original signal [42]. Some other groups proposed wave-front shaping methods to focus the light passing through a multimode fiber. Even though these methods successfully focused the light, they required continuous recalculation of the optimal wave due to the fiber motion [39,42]. These methods do not work in the case of reflection mode detection of objects. In reflection mode imaging, the transmission matrix describing the response between the modes at the input and output planes can be used to overcome the distortion [39].

The modal dispersion effect is avoided using GRIN fibers, where the refractive index change along the section of the fiber equalizes the travel time of different modes. Thus, different spatial modes propagate at similar velocities. Sato et al. used a GRIN fiber for the fabrication of a single-fiber endoscope used for reflectance imaging. However, this device had some problems related to nonuniform image quality, background distortion, etc. [43]. High-quality photoacoustic images using a GRIN fiber are reported in [44], where the light focusing property of the GRIN fibers permitted the propagation of spatially distributed Gaussian beams through the fiber, which enhanced the focusing of the spot at the output. This, in turn, permitted high-resolution imaging [44].

Double-clad fibers consisting of a central core and two outer cladding layers are another type of frequently used fibers in endoscopes. These fibers possess the unique feature of allowing the propagation of both single-mode and multimode light through the fiber. The single-mode light travels through the central core, while the multimode light is transmitted through the inner cladding material. Such a fiber is principally used in fluorescence imaging devices having single-mode illumination and multimode signal collection. Thus, the advantages of single-mode illumination and multimode collection are combined in these fibers [45,46,47].

It is possible to combine OCT and fluorescence imaging in a single endoscope using a DCF. In this case, OCT illumination and fluorescence excitation light is projected on the sample through the core of the DCF. The backscattered OCT signal is collected through the core, and the fluorescence emission from the sample is collected through the inner cladding of the fiber. The OCT source light and fluorescence excitation light are combined using a wavelength division multiplexer (WDM) before sending it through the core of DCF. The recollected light is separated using a DCF coupler, where the recollected OCT signal from the core of the DCF is submitted to an SMF, while the fluorescence signal from the inner cladding is forwarded to an MMF [48,49].

Buenconsejo et al. developed a device that combined narrowband reflectance, OCT, and autofluorescence imaging in a single-fiber endoscope using a DCF. This device worked analogously to other OCT devices, except for the difference that the red/green/blue (RGB) light was emitted from the central core, while the collection of the reflected light was performed through the inner cladding. The separation of the various light signals from three modalities was done using an additional WDM [50].

**Figure 2:**
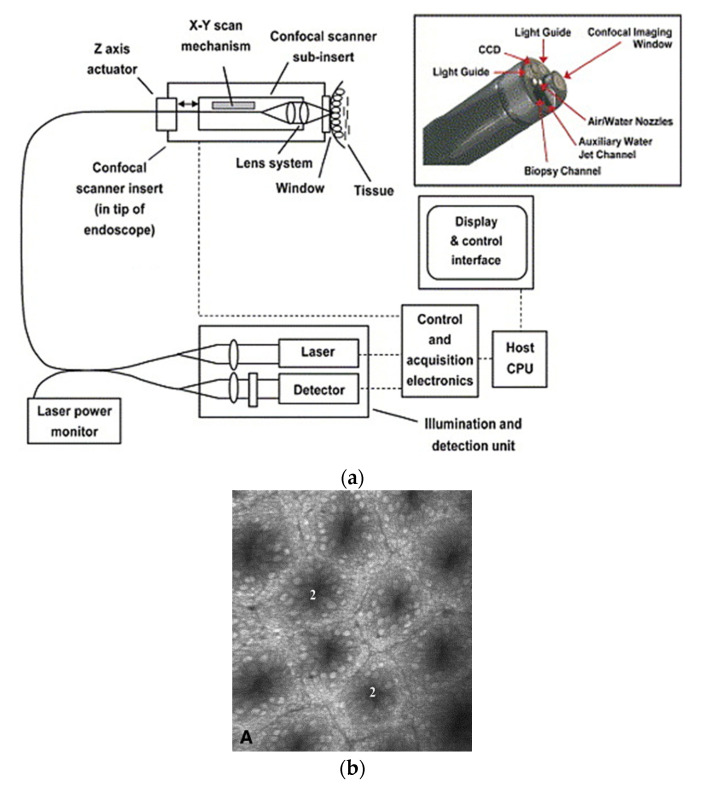
Fluorescence confocal imaging developed by Pentax: (**a**) schematic design of the micro-endoscope; (**b**) an en face image of rectal mucosa showing the crypt lumens (taken with the permission of [73]).

In a single-fiber-based micro-endoscope, the light beam can be steered at either the proximal or distal tip of the fiber. The possibility of using a light beam for a proximal scan allows the separation of large-sized beam scanning devices from the distal end of the endoscopic device used to monitor the target sample. Thus, it is possible to develop small-sized endoscopic devices that can image the deep tissue systems within the body. Proximal scanning is usually performed using side-viewing imaging probes, where a drive mechanism rotates the fiber to scan the light beam along the circumference of the target sample [51]. On the other hand, distal scanning is preferentially used in cantilever-based single-fiber endoscopes where the fiber tip is displaced mechanically using a variety of actuators. Usually, the fibers are excited at resonance to obtain high tip displacements using piezoelectric [52,53,54,55,56,57,58,59,60], electrostatic [61,62], electromagnetic [63,64], electrothermal [65,66,67], micromotor mirror [68,69,70], or shape memory alloy [71] actuators. The working principle of these actuators will be discussed in detail later in the paper. Pan et al. developed a fiber optic scanner where the beam was steered at the distal end using a pair of micromirrors [72]. The only commercially available single-fiber endoscope was developed by Pentax to image the upper and lower gastrointestinal (GI) tract. The optical scheme of this device is shown in Figure 2a [73]. In this case, the confocal images showing the subcellular and cellular structures of the upper and lower GI tract are imaged after the administration of the contrast agent. An in-vivo confocal image of rectal mucosa in human colon collected using the Pentax endoscope is shown in Figure 2b, where crypt lumens can be clearly identified [73].

### 2.3. Graded-Index (GRIN) Lens Scanner

In lens scanners, light is deflected due to a non-planar interface between the air and the lens. The lateral motion of the lens perpendicular to the direction of the incident beam in these scanners causes variation in the refraction angle, allowing light scanning.

**Figure 3:**
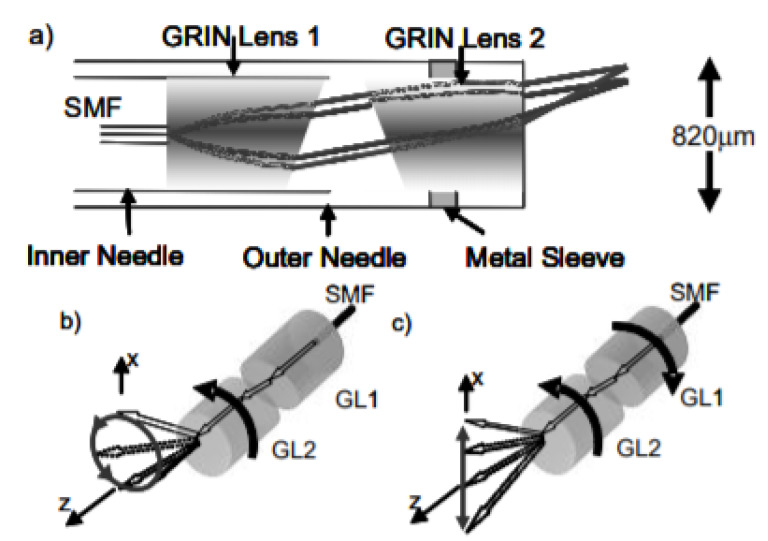
Schematic diagram of a PARS-OCT probe: (**a**) distal tip; (**b**) circular motion generation by rotating just one lens; (**c**) linear scan by rotation of both lenses (taken with permission of [74]) © The Optical Society.

**Figure 4:**
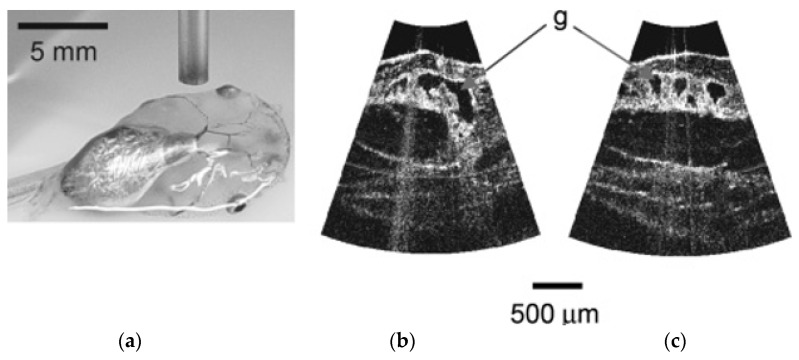
Endoscopic OCT image of the gill structure of a tadpole: (**a**) photograph of probe relative to the tadpole; (**b,c**) OCT images showing gill pockets indicated with g (taken with the permission of [74]). © The Optical Society.

Wu et al. developed a paired-angle rotating scanning OCT (PARS-OCT) probe to image the gill structure of a Xenopus laevis tadpole, where the beam steering the distal end of the probe was obtained by the rotary motion of the two angle polished GRIN lenses. A schematic diagram of this system is shown in Figure 3 [74]. The OCT images of the gill structure of a tadpole obtained using this device are shown in Figure 4. The photograph of an OCT probe relative to the tadpole is shown in Figure 4a, while the OCT images in Figure 4b,c enable to clearly identify the gill pockets [74]. Sarunic et al. integrated a gear-based linear scan mechanism with the PARS-OCT device to control the rotational speed of inner and outer GRIN lenses. They were able to identify vitreous, retina, and choroid surfaces in the OCT images of an ex vivo porcine retina [75].

## 3. Cantilever Beam Mechanics

In small cantilevered optical scanners, the image is obtained by scanning the light beam at the distal end of the device, as stated earlier. In most of the earlier endoscopes using CFBs to transport the light to the tissue surface, the beam is scanned using micromirrors placed at the proximal end of the device. In these so-called proximal scanners, the large-dimensioned scanning components can be separated from the distal end of the endoscopic device. Thus, it is possible to fabricate very compact-sized scanning devices. However, as the beam sweeps light across the CFB, a portion of the light enters through the cladding of the fibers as well, which results in poor contrast in the image. On the other hand, the scanning device is placed at the distal end of the endoscopic scanner to illuminate the light on the target sample in distal scanners. These single-fiber-based endoscopes require distal scanning to sweep the light across the target sample [24].

Among the distal scanners, it is possible to have two different configurations of devices based on the scanning direction [6]. In a side-view imaging device, the light from the fiber tip is deflected at a certain angle with the help of reflecting mirrors, or prisms. Such imaging devices provide circumferential images of the target surface, and 2-D cylindrical images can be obtained by moving the device along its axis [76]. However, in the forward-view imaging devices, the laser light is laterally scanned using special actuators and provides the image of the tissue surface at the front of the device [77].

The cantilever-based endoscopic scanners belong to the category of forward-view imaging devices. In such devices, an optical fiber is fixed at a distance of a few millimeters from its distal end. The free end of the fiber acts as a cantilever beam, which is vibrated, using certain actuators described later in the paper, to illuminate the target tissue area. The backscattered reflected light is used to reconstruct the image of the area using certain image processing algorithms. These cantilevered optical fibers can be vibrated in resonance, or at a frequency different from their resonant frequency.

Almost all the cantilevered-fiber optic endoscopes can be considered as cylindrical-shaped beams. The first resonant frequency (also called natural frequency) of a cylindrical-shaped cantilevered beam (where one side is rigidly blocked for any movement and the other end is free to move) is given by:

(6) $$f_{n}=\frac{1.875^{2}}{4\pi }\sqrt{\frac{E}{\rho }}\frac{R}{L^{2}}$$

with *E*, *ρ*, *R*, and *L* being the Young’s modulus, density, radius, and length of the cantilever beam, respectively [78]. From this equation, the driving frequency in resonant scanners depends on the inherent properties and dimensions of the optical fiber acting as the cantilever beam. In nearly all such scanners, the fiber is a standard 125 µm diameter fiber. Thus, the resonant and driving frequencies can be adjusted by changing the length of the cantilevered section.

**Figure 5:**
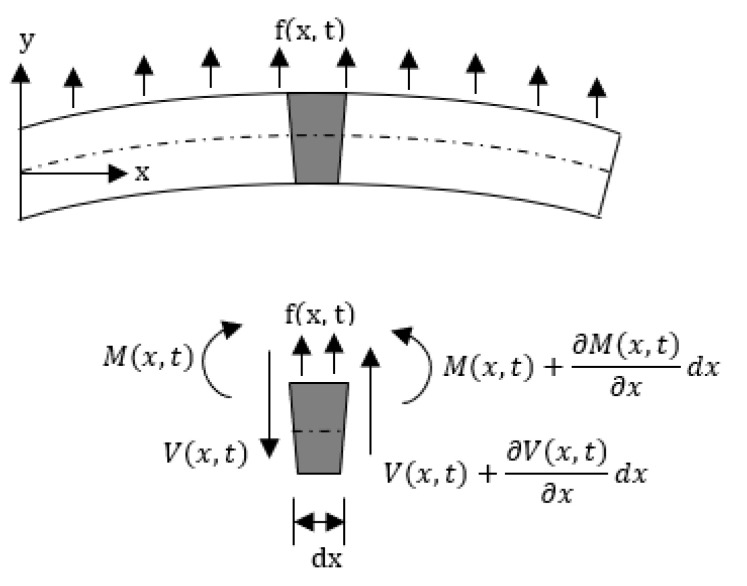
Euler–Bernoulli beam and a free body diagram of an element of the beam.

The deflection of a cantilevered beam in the transverse direction can be obtained considering the Euler–Bernoulli beam. The Euler–Bernoulli beam theory describes the relationship between the beam deflection *w*(*x*,*t*) and the applied load *f*(*x*,*t*), assuming small deformations in the beam such that the planes perpendicular to the *x-y* axis do not bend after the deformation. The equation describing the deflection *w*(*x*,*t*) of the beam in the y direction, in time (*t*) and along the length (*x*), can be derived considering the force and moment equilibrium of an infinitesimal element *dx* of the beam as in Figure 5. The equilibrium of forces in the *y* direction yields:

(7) $$\left(V\left(x,t\right)+\frac{\partial V\left(x,t\right)}{\partial x}dx\right)-V\left(x,t\right)+f\left(x,t\right)dx=\rho A\left(x\right)dx\frac{\partial^{2}w\left(x,t\right)}{\partial t^{2}}$$

where *V(x,t)* is the shear force and *f*(*x*,*t*) the total applied external force per unit length, while the term on the right-hand side describes the inertial force of the element, with *A*(*x*) being the cross-section of the beam. Similarly, the equilibrium of moment acting on the element can be written as:

(8) $$\left[M\left(x,t\right)+\frac{\partial M\left(x,t\right)}{\partial x}dx\right]-M\left(x,t\right)+\left[V\left(x,t\right)+\frac{\partial V\left(x,t\right)}{\partial x}dx\right]dx+\left[f\left(x,t\right)dx\right]\frac{dx}{2}=0$$

with *M*(*x*,*t*) being the bending moment related to beam deflection *w*(*x*,*t*) and flexural stiffness *EI*(*x*) of the cantilever beam, where *E* is the Young’s modulus, and *I*(*x*) is the cross-sectional area moment of inertia [78]. *M*(*x*,*t*) is given by:

(9) $$M\left(x,t\right)=EI\left(x\right)\frac{\partial^{2}w\left(x,t\right)}{\partial x^{2}}$$

Simplifying and neglecting higher order terms in Equation (8), and combining it with (7) and (9), gives:

(10) $$\rho A\left(x\right)\frac{\partial^{2}w\left(x,t\right)}{\partial t^{2}}+\frac{\partial^{2}}{\partial x^{2}}\left[EI\left(x\right)\frac{\partial^{2}w\left(x,t\right)}{\partial x^{2}}\right]=f\left(x,t\right)$$

The beam deformation under free vibration can be attained by considering *f(x,t)* = 0. For beams with a uniform cross-section, Equation (10) can be further simplified by having *A*(*x*) = *A*, and *I*(*x*) = *I*. Thus,

(11) $$\frac{\partial^{2}w\left(x,t\right)}{\partial t^{2}}+\frac{EI}{\rho A}\frac{\partial^{4}w\left(x,t\right)}{\partial x^{4}}=0$$

The beam deflection can be solved using this equation with four boundary conditions and two initial conditions. The initial conditions are the specified initial deflection $w_{0}\left(x\right)\;$ and velocity ${\dot{\omega }}_{0}\left(x\right)$ profiles causing the motion:

(12) $$w\left(x,0\right)=w_{0}\left(x\right)\;and\;w_{t}\left(x,0\right)={\dot{\omega }}_{0}\left(x\right)$$

For a cantilever beam, the boundary conditions are the zero bending moment and the shear force at the free end, and no deflection and slope at the fixed end. In other words,

(13) w(0,t)=0

(14) $$\frac{\partial w\left(0,t\right)}{\partial x}=0$$

(15) $$EI\frac{\partial^{2}w\left(0,t\right)}{\partial x^{2}}=0$$

(16) $$\frac{\partial }{\partial x}\left[EI\frac{\partial^{2}w\left(0,t\right)}{\partial x^{2}}\right]=0$$

**Figure 6:**
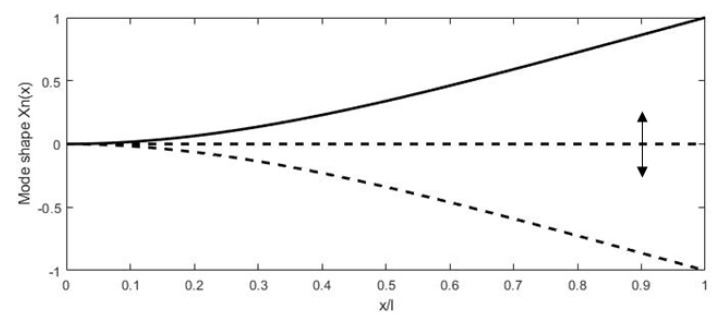
Deformation of a cantilever beam at resonance.

Equation (11) can be solved by separating variables as in *w*(*x*,*t*) = *X*(*x*)*T*(*t*). This approach permits the separation of Equation (11) into two sub-equations, which can be solved separately to yield temporal and spatial results. The total solution can be obtained by combining the two results. As stated above, the temporal solution depends upon the initial conditions, which vary from case to case. Given the boundary conditions, the spatial part yields:

(17) $$X\left(x\right)=\cosh\left(\beta_{n}x\right)+\cos\left(\beta_{n}x\right)+\sigma_{n}\left[\sinh\left(\beta_{n}x\right)+sin\left(\beta_{n}x\right)\right]$$

where $\beta_{n}$ and $\sigma_{n}$ are the coefficients depending on the mode considered. For the first resonant mode, $\beta_{n}l$ is 1.875, while $\sigma_{n}$ is 0.7341 [78]. The first mode shape of a cantilevered beam actuated at resonance is shown in Figure 6 along with the beam in an initial state.

### 3.1. Resonantly Actuated Scanners

In scanning fiber endoscopes, cantilevered fibers are usually excited at resonance to scan the light beam. The main advantage of using the driving frequency equal to the resonant frequency consists in obtaining a higher tip displacement of the free end of the fiber, which results in high-resolution images (higher number of pixels in the FOV). It is possible to excite the cantilevered fiber using a variety of micro-actuators, such as piezoelectric [51,79,80,81], electromagnetic [82,83,84], electrothermal [85,86,87], shape memory alloys [88], or electroactive ionic polymer [89] actuators. The distal end of the fiber follows the mode shape shown in Figure 6. Each actuation method is better suited for the excitation frequency in certain ranges based on their working principle, which will be described in detail later in the paper. Various resonant scanners in the literature are compared in [90].

Depending upon the actuation technique used to excite the cantilever beam, it is possible to observe the development of 2D motion of the fiber tip by exciting the fiber along one direction. The so-called whirling motion, causing the fiber tip to follow an elliptical-shaped pattern instead of its linear motion, is caused by the cross-coupling of the motion between the planes perpendicular to the beam axes. It is possible to obtain a stable whirling motion within a small frequency range [91,92]. The cross-coupling motion can be avoided by exciting the cantilever beam along certain eigendirections [93]. On the other hand, Wu et al. developed a fiber optic scanner able to obtain 2D scanning using nonlinear cross-couplings [94].

### 3.2. Non-Resonantly Actuated Scanner

Some actuation methods such as electrothermal actuators are unable to generate motion at very high frequency. It is difficult to generate resonant scanners characterized with a low resonant frequency as they require long and slender beams, compromising their mechanical stability. In addition, the fiber tip displacement occurs symmetrically to the optical axis and is difficult to offset in resonant scanners. In such cases, the cantilevered optical fibers are excited at a frequency different from their resonant frequency. Even though there is less tip displacement for a given excitation power, it is possible to achieve beam scanning at low frequency and offset the center of the scan by adding a bias voltage [90].

In such scanners, the deflection of the distal tip of the fiber $\left(\delta_{tip}\right)$ is related to the displacement of the actuator exciting the vibration $\left(\delta_{s}\right)$ by:

(18) $$\delta_{tip}=\delta_{s}\left[1+\frac{3\left(1-a\right)}{2a}\right]+\frac{qgL^{4}}{8EI}$$

with *L*, *a*, *q*, *E*, *I*, *g* being the length of the cantilevered portion of the fiber, length ratio of the fixed end, mass per unit length of the fiber, Young’s modulus, the moment of the inertia of the fiber, and acceleration of the gravity, respectively [95].

Zhang et al. proposed a similar scanner where a 45-mm-long fiber was electrothermally actuated using a micro-electro-mechanical (MEMS) actuator operating at no more than 6 V [95]. A similar scanner was developed by Park et al., where a 40-mm-long fiber, used as an endoscopic OCT probe, was actuated using a 3 V power source [96]. Some researchers were able to develop cantilevered scanners working at a frequency not too far from their resonance frequency. In such semi-resonant scanners, the fiber tip provided an intermediate displacement, and no nonlinear whirling effects were present. Moon et al. developed an OCT probe where the cantilever scanner was excited at 63 Hz using a piezo-tube actuator [97].

## 4. Actuators in Cantilever-Based Endoscopic Devices

The miniature size of MEMS devices, along with their light weight and stable performance characteristics, makes them attractive for micro and nano applications, among which are endoscopic optical devices. On the basis of the working principle, MEMS actuators can be subdivided into piezoelectric, electrostatic, electrothermal, electromagnetic, and shape memory alloy actuators. The piezoelectric actuators are widely used in endoscopic catheter design due to their compact size, low power consumption, and large output force. On the other hand, the actuation displacement is limited in such devices. Electrostatic actuators are the second most used actuation method in medical scanning devices due to their fast response and ease of fabrication. However, it is difficult to produce such devices at very small dimensions, which limits their use in systems requiring a distal actuation. Electrothermal actuators generate high actuation displacement and force, but the elevated working temperature and low working frequency limit their use in some cases. Electromagnetic and shape memory alloy actuators find limited applications in cantilevered fiber optic endoscopic scanners [98]. All these actuation methods are described in this section in great detail and compared in Table 2, where the number of ticks qualitatively indicates the intensity of a certain pattern [6].

### 4.1. Piezoelectric Actuators

The working principle of piezoelectric actuators is based on the so-called piezoelectric effect. Piezoelectric materials have the ability to change the material polarization in the presence of a mechanical stress and conversely generate strain or force in the presence of an external electric field. Among the various crystalline, ceramic, and polymeric materials, aluminum nitride (AlN) and lead zirconate titanate (PZT) are most frequently used in MEMS devices [99]. Piezoelectric actuators are characterized by providing fast response, low driving voltage, and low power consumption [100].

The relationship between the electric field applied to the material and the mechanical deformation exhibited by the material is nonlinear due to the presence of hysteresis and drift. For a small variation in electric field, the material behavior is almost linear and can be described by:

(19) $$\varepsilon =Ed+c^{-1}\sigma$$

where *ε* is the strain tensor, *E* is the electric field vector, *σ* is the stress tensor, *d* is the piezoelectric tensor (vector of strain coefficients), and *c* is the elastic tensor. In the case of no external force, the second component on the right-hand side of Equation (19) becomes zero. The piezoelectric strain depends upon the direction of the mechanical and electrical fields [101].

Piezoelectric materials often show a nonlinear hysteresis behavior, which causes the relationship curve between the displacement exhibited by the material and the applied electric field to be different in ascending and descending directions. Various models have been proposed to describe this hysteresis phenomena. Bahadur and Mills proposed a hysteresis model to characterize the symmetric and asymmetric rate-dependent hysteresis. The output charge (*q*) is related to the endpoint displacement (*x*) and the applied voltage (*V_p_*) by:

(20) $$q=C_{0}V_{p}+T_{em}x$$

with *C*_0_ and *T_em_* being the capacitance of the piezoelectric element and the electromechanical coupling factor, respectively [102].

Normally, piezoelectric devices are restricted for 1D operation with force/displacement occurring along the axis defined by the electric field. In these so-called longitudinally translating piezo chips, the electric field is applied parallel to the polarization direction of the material, which causes the displacement in the same direction of the field normal to the surface of the electrodes. In shear piezo elements, the polarization is obtained in the direction perpendicular to the field direction. Thus, there is an orthogonal relationship between the direction of the displacement and that of the electric field [103].

Piezoelectric actuators are available in single disc/plate and tubular configurations. These configurations are described below in detail.

#### 4.1.1. Disc Piezoelectric Actuator

**Figure 7:**
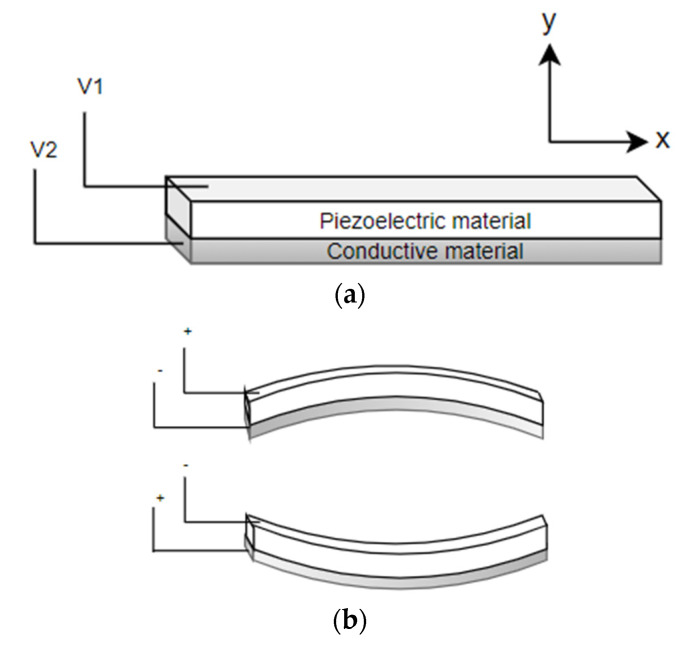
Piezoelectric bending actuator: (**a**) schematic diagram; (**b**) working principle.

Flat disc piezoelectric actuators can be constructed using a single piezo element (unimorph actuators) or using two different piezo elements (bimorph actuators). In either case, piezo elements are connected to a base material. There is an expansion or contraction of the piezo material in the presence of an electric current, which provides the bending motion of the actuator. The schematic diagram showing the working principle of a piezo bending actuator is shown in Figure 7. When a positive voltage is applied to the piezoelectric ceramic layer, it elongates in x direction, while the base material does not change its length, resulting in convex bending of actuator towards the conductive layer, as in Figure 7b. Similarly, bending in the opposite direction occurs by applying a negative voltage to the piezoelectric sheet. Li et al. fabricated a scanning fiber probe for an OCT endoscope, where an optical fiber was placed in the middle of the two piezoelectric plates with a common copper substrate element. The bending of the fiber tip in the vertical direction was obtained by exciting the two piezo elements with the same voltage, i.e., the structure will bend along the positive or negative directions by elongating or contracting both elements. On the other side, the motion of the tip along the horizontal axis was obtained by applying an opposite polarity voltage to the two elements. A two-dimensional Lissajous scanning pattern was obtained by the fiber tip by controlling the voltages on the two layers [57].

**Figure 8:**
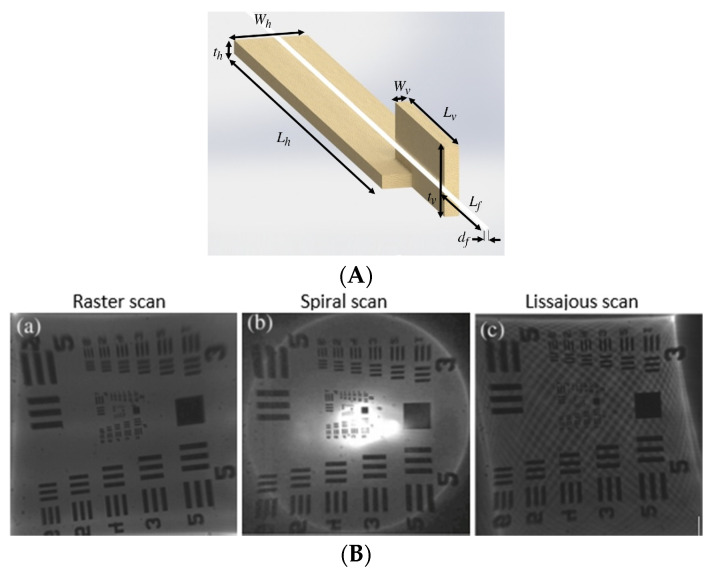
Multiple patterns generating fiber scanner: (**A**) schematic diagram of the scanner; (**B**) achieved scanning patterns projected on a USAF target (taken with permission of [104]).

Tekpinar et al. developed a piezoelectric fiber scanner where two planar piezo bimorph cantilever actuators were placed perpendicular to each other to generate the 2D motion of an optical fiber connected to them. In this case, the dimensions of the cantilevered fiber and the mode optimization allowed the fiber tip to interchangeably follow a raster, spiral, or Lissajous scanning pattern by simply changing the actuation parameters. A schematic diagram of such a model is shown in Figure 8A [104]. The different scanning patterns were projected on a United States Air Force (USAF) resolution target to evaluate the image uniformity. From Figure 8B, it can be seen that a raster scan provides a uniform illumination on the target ((a) within Figure 8B), while the spiral one is characterized by decreasing illumination along the radius ((b) within Figure 8B). The illumination in the case of a Lissajous scan is highly dependent on the fill factor ((c) within Figure 8B) [104].

**Figure 9:**
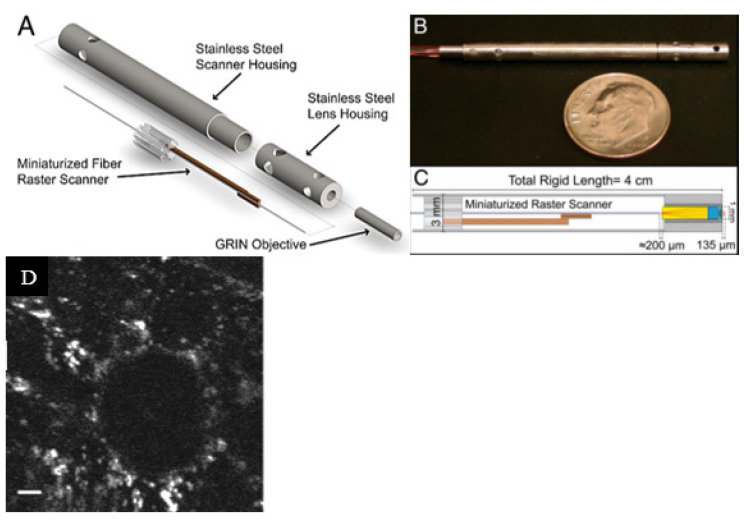
Raster scanning endoscope: (**A**) mechanical assembly; (**B**) developed prototype; (**C**) optical path diagram; (**D**) fluorescence image of an unstained ex vivo lung tissue (taken with the permission of [105]).

Rivera et al. developed a compact multiphoton endoscope (with an outer diameter of 3 mm) where two bimorph piezoelectric actuators were used to excite a DCF. Two bimorph structures were placed in such a configuration to have perpendicular bending axes. A raster scanning pattern was generated by exciting the fiber in two directions. The fast-scanning motion was obtained by exciting one of the actuators at the resonant frequency of the extending cantilevered DCF, and slow axis scanning was performed by exciting the other actuator at a frequency much lower than the resonance frequency. The mechanical assembly of the described endoscope is illustrated in Figure 9a [105]. The developed prototype and the optical path diagram inside the endoscopic tip are shown in Figure 9b,c [105]. Using the developed probes, the authors were able to obtain the fluorescence images of an ex vivo mouse lung tissue at depths comparable to a commercial multiphoton microscope. The finer resolution (lateral and axial resolution of 0.8 and 9.4 µm, respectively) enabled the probe to clearly identify the alveolar walls and lumens in the unstained lung tissue, as in Figure 9d [105].

It is possible to enhance the cantilever fiber deflection with low power consumption by using very thin piezoelectric ceramic layers. Recent developments in the field showed the ability to form piezoelectric layers with thicknesses at submicron levels. A variety of methods were implemented to deposit the thin film of these materials. Such methods included arc discharged reactive ion-plating, epitaxial process, sol–gel spin-coating, and sputtering [100].

#### 4.1.2. Tubular Piezoelectric Actuator

In the tubular structure, a thin ceramic sheet is bent into a cylindrical shape and can experience axial, radial, or bending motion. The tube shrinks radially and axially in the presence of a voltage difference between the inner and outer electrodes of the tube. In these actuators, the load can be mounted either on the curved surface for radial displacement or on the rims for axial displacement. These devices are very rapidly responsive, but the generated force is limited. Usually, the displacement produced by a piezoelectric device is very small. Thus, it is possible to stack up various piezoelectric disks or tubes to amplify the generated displacement [99].

**Figure 10:**
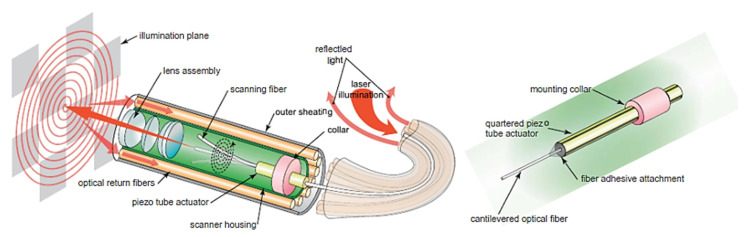
Schematic diagram of a scanning fiber endoscope (taken with permission of [15]).

**Figure 11:**
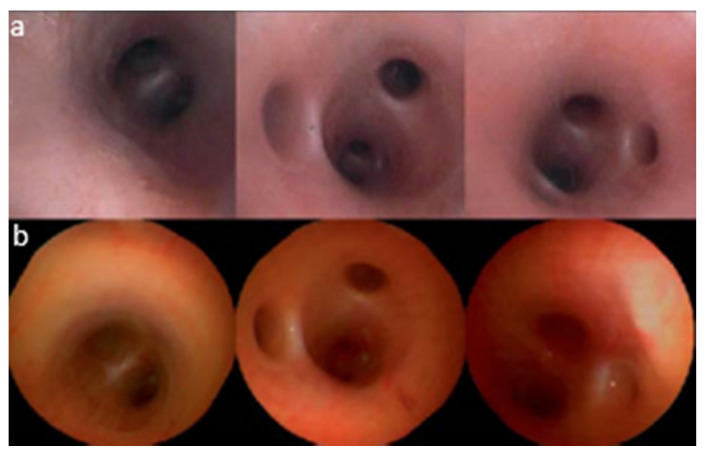
In vivo images of airways of a pig acquired with (**a**) conventional Pentax bronchoscope; (**b**) scanning fiber endoscope (taken with permission of [15]).

As described earlier, piezoelectric actuators are most commonly used in cantilever-based endoscopic probes, especially the tubular piezoelectric actuators. In this case, the tube structure is divided into four electrodes and placed near the blocked end of the cantilever fiber. The base excitation of the fiber along a certain direction is obtained by applying voltage to two opposite electrodes. Seibel et al. used this configuration to obtain a 2D displacement of the fiber tip. In this small-sized endoscope, the drive voltage at two pairs of electrodes had an increasing amplitude and a phase shift of 90°, which resulted in a spiral pattern followed by the fiber tip [9,10]. The schematic diagram of the scanning fiber endoscope is shown in Figure 10 along with an enlarged view of the scanning portion showing the cantilevered fiber’s connection with the tubular actuator [15]. Some in-vivo testing images of airways of a live pig taken with this endoscope (Figure 11b) were compared with the corresponding images from a conventional Pentax bronchoscope (Figure 11a). The two devices showed comparable images in terms of resolution and field of view [15].

**Figure 12:**
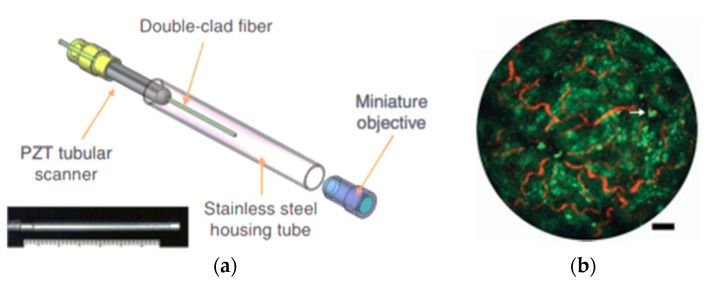
Nonlinear optical endoscope: (**a**) schematic diagram; (**b**) two-photon and second harmonic generation structural image of a resected mouse liver (taken with permission of [106]).

A similar fiber optic scanner using a tubular piezoelectric actuator, packaged within a 2 mm housing tube, was developed by Liang et al. as a two-photon and second harmonic endoscope. This novel endo-microscope enabled label-free histological imaging of tissue structures with subcellular resolution. The schematic diagram of the developed two-photon endoscope is illustrated in Figure 12a [106]. An overlaid two-photon and second harmonic generation image of a mouse liver acquired with such an endoscope is shown in Figure 12b, where the collagen fibers (in red) and vitamin A granules (in bright green) dispersed in the cytoplasm (dark green) can be clearly identified [106].

**Figure 13:**
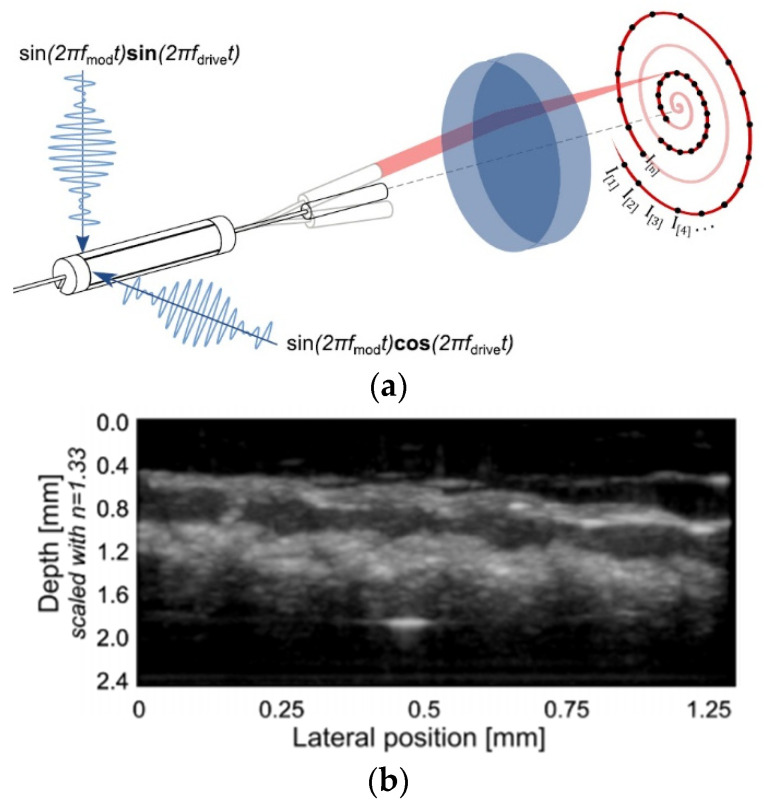
Fourier-plane fiber scanner: (**a**) schematic diagram; (**b**) cross-sectional tomogram of human finger (taken with permission of [107]).

Vilches et al. developed a fiber scanner for OCT where a piezoelectric tubular actuator provided the base excitation motion to an optical fiber having a GRIN lens attached to its free end. In this configuration, schematized in Figure 13a, the beam scanning was attained by rotating the angle of the collimated beam, which provided high-resolution imaging while avoiding optical aberrations [107]. The cross-sectional tomogram of a human finger obtained with this scanner is shown in Figure 13b [107].

### 4.2. Electrothermal Actuators

The working principle of an electrothermal actuator is based on the Joule effect. The electric resistivity causes an increase in temperature in the presence of the current flow through the actuator. The amount of heat generated in a material is directly proportional to the material’s resistivity, current, and the length of the actuator, while it is inversely proportional to the cross-sectional area of the device. Generated heat causes thermal expansion and consequently the deformation of the material.

In electrothermal actuators, the cross-section is usually much smaller than the length of the actuator to make it more resistive and, consequently, cause higher temperature variations for a given input power. Thus, the temperature along the actuator can be calculated using a one-dimensional model. A correction factor can be included in the equation to consider this approximation. The nonlinear partial differential equation describing the temperature variation (*T*) in space and time can be obtained using the conservation of energy [108]. In the case of a rectangular section bar (with width *w*, and height *h*), the partial differential equation (PDE) describing the heat transfer along the length x becomes:

(21) $$\rho c_{p}\frac{\partial T}{\partial t}dx=J^{2}\rho_{r}dx-\left[\frac{d}{dx}\left(-k_{p}\frac{dT}{dx}\right)dx+\left(T-T_{p}\right)\left(\frac{S}{hR_{T}}\right)dx+\left(\frac{2h_{cs}}{w}+\frac{\lambda h_{cf}}{h}\right)\left(T-T_{a}\right)dx+\frac{\lambda \varepsilon_{x}\sigma }{h}\left(T^{4}-T_{a}{}^{4}\right)dx\right]$$

with *ρ*, *c_p_*, ρ_r_, *k_p_* being the density, specific heat, resistivity, and the thermal conductivity of the material characterizing the actuator, respectively. *h_cs_* and *h_cf_* are the convection coefficients for the side walls and the faces of the actuator element, respectively. *λ* is the coefficient describing the heat loss. *ε_x_* and *σ* are the surface emissivity and the Stefan–Boltzmann constant for radiation heat transfer, respectively, while *T_p_* and *T_a_* are the substrate and ambient temperature, respectively. *R_T_* is the thermal resistance between the actuator surface and the substrate material. *J* is the current density along the actuator given by the current passing through it per unit section of the actuator material. *S* is the shape factor and is a function of total heat flux defined as:

(22) $$S=\frac{h}{w}\left(\frac{2t_{v}}{h}+1\right)+1$$

where *t_v_* is the air gap between the actuator material and the substrate [108,109].

The heat transfer through convection and radiation is evident at very high temperatures. In electrothermal actuators, the operable temperature is limited to avoid damage to the material. Thus, the corresponding terms in Equation (21) can be neglected [108,110]. Therefore, Equation (21) can be simplified to:

(23) $$\rho c_{p}\frac{\partial T}{\partial t}=J^{2}\rho_{r}+k_{p}\frac{d^{2}T}{dx^{2}}-\left(T-T_{p}\right)\left(\frac{S}{hR_{T}}\right)$$

The temperature profile along the actuator can be determined knowing the initial temperature of the actuator and the two boundary conditions.

On the basis of configuration, electrothermal actuators can be divided into hot-and-cold arm, chevron, and bimorph actuators.

#### 4.2.1. Hot-and-Cold Arm Actuators

These actuators are also called U-shaped actuators, folded beam actuators, heatuators, or pseudo bimorph actuators. As the name implies, the structure of the actuator is made up of at least one hot arm and one cold arm. The actuator is usually made up of a homogenous material with folded arms in a U-shaped pattern that are constrained by anchors. Usually, the anchor surfaces are characterized by having a large surface area, required to ensure heat dissipation. Two arms of the actuator characterized by different cross-sections are connected in series to an electric circuit. The current flows through the structure with different current densities within the two arms. Therefore, more heat is produced within the thin arm through the Joule heating principle compared to that of the wide arm. This differential thermal expansion of the material causes the thin arm to expand more and bend towards the wide section, generating the bending moment [98,111].

At the base of the cold arm, there is a thin section flexure arm which helps the bending deflection at the tip of the actuator in the shape of an arc in the actuator plane. The length of the flexure arm plays an important role in the value of the tip deflection. In the original model proposed by Guckel at al., the length of the flexure arm and the wide arm were equal to half of the length of the thin arm [112]. Huang and Lee developed the mathematical model describing the tip displacement of the actuator tip with respect to the function of the air gap between the two arms and the geometry of the arm structures. The smaller gap between the two arms led to a higher tip displacement, and the optimal length for the flexure arm was around 14–18% of the total length of the thin arm [113].

The temperature along the beam can be obtained by unfolding the beam and applying Equation (23). In the case of no external load acting on the tip, the lateral deflection of the tip at the free end of these actuators can be described by:

(24) $$\delta y=\frac{1}{2}\frac{\left(a^{4}-a^{2}+2a\right)Ar\alpha \Delta T_{net}L^{2}}{5a^{4}I+a^{4}r^{2}A-2a^{3}I+5aI+r^{2}aA+I+a^{5}I-2a^{2}I}$$

where *L*, *a*, *A*, *r*, *α*, *I* are the actuator length, ratio of flexure arm length to hot arm length, cross-section area of the flexure and hot arm, center gap between the hot arm and the flexure component, coefficient of thermal expansion, and the moment of inertia of the hot arm (flexure arm), respectively. Δ*T_net_* is the net temperature difference defined as the temperature which would cause the expansion of the hot arm alone and is the same as the net expansion in the real actuator case, where a small expansion of the cold arm corresponds to a decrease in the flexure component and results in a decrease in the net expansion between the two arms [114].

Hot-and-cold arm actuators are used widely in MEMS devices. There are large variations in the geometry of the actuators to achieve asymmetric thermal expansion. It is possible to obtain the in- plane deflection by changing the length of the arms instead of the cross-section [115], or using a combination of both the difference in the length and cross-section [116], or connecting the two arms of the actuator in parallel instead of series, enabling higher current density in the thick arm, causing the tip deflection towards the thin arm [117], or changing the resistivity of one arm by selectively doping it.

**Figure 14:**
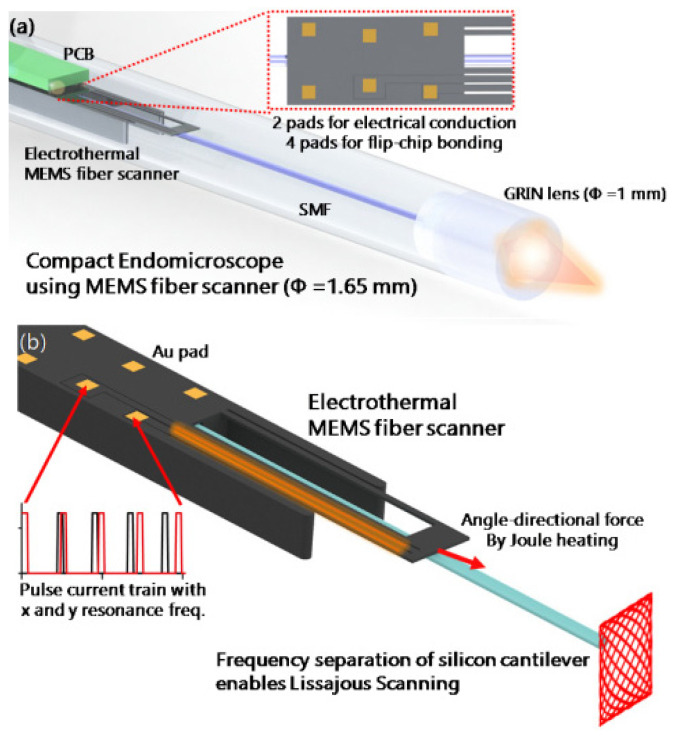
Electrothermally actuated confocal endo-microscope: (**a**) schematic diagram; (**b**) working principle (taken with permission of [65]).

Lara-Castro et al. designed an array of four electrothermal actuators based on the hot-and-cold arm configuration to obtain out-of-plane displacement. Four actuators are used to control the rotation of a MEMS mirror for endoscopic OCT purposes [118]. Some other changes in the geometry of the actuator include using two hot arms [66,119]. Seo et al. used this kind of double hot arm electrothermal actuator to obtain the lateral in-plane displacement of the optical fiber for endoscopic purposes [66,120]. An optical fiber was firmly connected to the linking bridge connecting the two hot arms and the cold arm. The differential thermal expansion between hot and cold arms allowed the cantilevered fiber to move in a lateral direction (in-plane motion), while that between the actuator surface and the fiber gave the vertical motion, causing the fiber tip to follow a Lissajous scanning pattern [66]. The schematic diagram of a 1.65 mm diameter confocal endo-microscope catheter developed using this type of actuator is shown in Figure 14 [65].

#### 4.2.2. Chevron Actuators

Chevron actuators are also called bent-beam actuators or V-shaped actuators and are the other type of in-plane electrothermal actuator, with a slightly different working principle. In this case, the in-plane displacement of the tip of the actuator is obtained from the total thermal expansion of the components instead of a differential expansion [98].

In a V-shaped electrothermal actuator, two symmetrical slanted beams are connected at a certain angle to a central shuttle beam at the apex to the base with anchors. The current passing through the actuator causes the thermal expansion of both slanted beams due to the Joule heating principle. As the movement of the beam is constrained by the anchors and the central shuttle, the thermal expansion causes a compression force and a bending moment, which gives rise to the lateral displacement of the shuttle beam [121].

Similar to the U-shaped beams, the temperature distribution along the arms of the actuator can be obtained using Equation (23). Enikov et al. described the analytical model for V-shaped thermal actuators. The analysis of the beam deformation was considered by taking into account the buckling effect in the beam due to the axial thermal load and transversely applied force, if any. The numerical solution of the thermoelastic buckling model of the beam led to the tip deflection of the beam or the central shuttle beam [121]. However, Sinclair presented a simplified model describing the tip displacement to be:

(25) $$\delta ={\left[l^{2}+2\left(l\right)l^{\prime }-l\cos{\left(\theta \right)}^{2}\right]}^{1/2}-l\;sin\left(\theta \right)$$

where *θ* is the initial tilt angle of the arm beam, *l* is the length of the single actuator arm, and *l*’ is the elongation due to thermal expansion [122].

These actuators provide certain advantages over the bent-beam actuators described earlier, such as rectilinear displacement, larger exhibited force at the tip, and lower power consumption [98]. The displacement of the central tip of the actuator can be increased by using longer arm components or reducing the bending angle *θ*. The opposite changes increase the exhibited force. Moreover, it is possible to amplify the motion of the shuttle beam by connecting the two bent-beam actuators in cascade. In the cascaded configuration, two V-shaped electrothermal actuators are anchored to the substrate and connected together with secondary V-shaped beams. The current can be passed either through the primary units only or through all the structural components [123]. Similarly, it is possible to increase the output force without changing the displacement from the device by placing multiple V-shaped actuators in parallel [122]. It is even possible to combine the parallel and cascade configurations to obtain the desired displacement and force outputs [124].

Another variation in chevron actuators consists of changing the geometry of the actuator to obtain a wide range of output properties. Among these, the most frequently used are the electrothermal actuators with Z-shaped patterned arms. In this configuration, the thermal expansion of the beams is blocked due to symmetry constraints, leading to the bending of the beams and thus the in-plane displacement of the central shuttle element. Z-shaped actuators permit smaller feature sizes and larger displacement compared to the V-shaped electrothermal actuators [98,125]. Another alternative in chevron actuators is a combination of straight and bent beams, or the so-called kink actuator. This kind of actuator consists primarily of straight arms which undergo thermal expansion by the Joule effect, while the small kink in the middle serves to guide the motion of the actuator. Kink actuators provide higher displacement at lower power levels as compared to V-shaped actuators [126].

**Figure 15:**
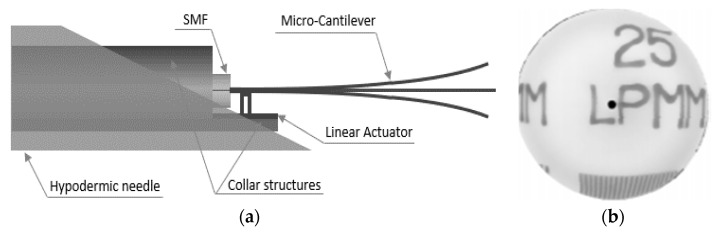
Cantilevered fiber scanner using chevron actuator: (**a**) schematic diagram; (**b**) reconstructed image of a resolution target.

Chevron actuators find use in some cantilever-based optical scanners. Kaur et al. developed a sub-millimeter-sized cantilevered fiber optical scanner that can find use as a forward-viewing endoscopic probe. In this design, shown in Figure 15a, an electrothermal chevron actuator made with two parallel legs excites an SMF at resonance. In this case, the total thermal expansion of the actuating material provides a base excitation motion to the cantilevered fiber [127]. A resolution target image captured with this scanner is provided in Figure 15b.

#### 4.2.3. Bimorph Actuators

A third class of electrothermal MEMS actuators is bimorph or bi-material type actuators. In this kind of actuator, two or more materials with different thermal expansion coefficients are stacked on top of each other. The different thermal expansion causes the actuator to bend or curl due to the induced strain generated by the Joule heating during actuation, which results in an out-of-plane motion [111].

The basic design of a bimorph electrothermal actuator consists of a cantilever-shaped micro-actuator fabricated using two layers of different materials connected to each other. The bending direction of the actuator tip during actuation will be dictated by the material with the higher thermal expansion coefficient compared to the one with the lower thermal expansion coefficient. The mathematical model for the tip deflection of such a micro-actuator is described by Chu et al. [128]. Assuming a constant curvature, the deflection at the free end of a cantilevered bi-material actuator is given by:

(26) $$\delta =kL^{2}/2\;$$

with *L* being the length of the cantilevered bi-metallic beam, and *k* being the curvature, which is:

(27) $$k=\frac{1}{r}=\frac{6b_{1}b_{2}E_{1}E_{2}t_{1}t_{2}\left(t_{1}+t_{2}\right)\left(\alpha_{2}-\alpha_{1}\right)\Delta T}{{\left(b_{1}E_{1}t_{1}{}^{2}\right)}^{2}+{\left(b_{2}E_{2}t_{2}{}^{2}\right)}^{2}+2b_{1}b_{2}E_{1}E_{2}t_{1}t_{2}\left(2t_{1}{}^{2}+3t_{1}t_{2}+2t_{2}{}^{2}\right)}\;$$

where *r* is the radius of curvature, *b*, *t*, *E*, *α* are the width, thickness, Young’s modulus of elasticity, and the thermal expansion coefficient, respectively, of the two materials characterizing the actuator. Δ*T* is the change in temperature due to Joule heating [128].

As with other electrothermal actuators, it is possible to place the different bimorph actuators in a cascaded configuration to amplify the obtainable tip displacement. In such structures, the various bimorphs are placed together in a serpentine direction, which causes the tip deflection from each bimorph to be added in series, yielding the higher overall tip displacement. It is possible to use different geometries for the bimorph structures to adapt according to the required spacing limitations in the microdevices. Large numbers of bimorph structures can be connected in a vertical cascaded form to generate a large out-of-plane displacement. Many bimorph structures can also be placed in parallel to lift the high load mirror surface [129].

**Figure 16:**
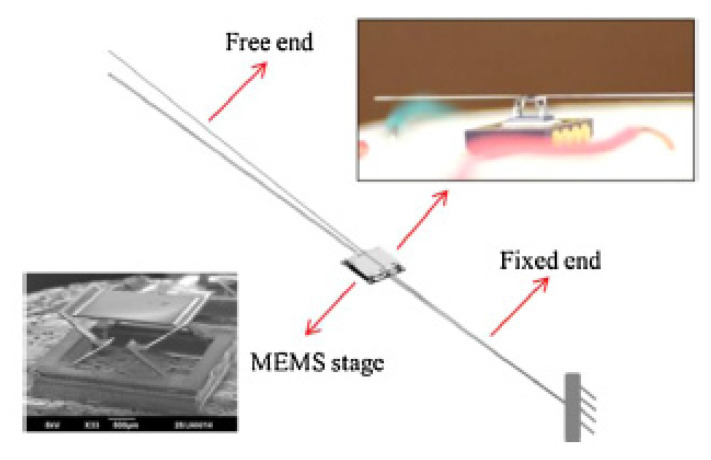
Schematic diagram of a non-resonant fiber scanner using bimorph electrothermal actuation technique (taken with the permission of [95]).

Bimorph actuators are the most frequently used electrothermal actuators and find use in a large number of scanning mirror MEMS devices. Zhang et al. developed a cantilevered fiber scanner excited non-resonantly using a MEMS stage. This platform, placed at a certain distance from the fixed end of the fiber, was connected to the fixed surface using three-segment bimorph actuators at four edges. Three segments of the Al-SiO_2_ bimorph actuators were placed in a configuration, shown in the schematic of Figure 16, to cancel out the lateral motion generating large vertical motion at the fiber tip [95]. Such a device along with imaging optics was packaged in a 5.5 mm probe to use as an endoscopic OCT probe [96].

### 4.3. Electromagnetic Actuators

The working principle that governs the motion in these actuators is the so-called electromagnetic principle, where the conversion from electric/magnetic energy to mechanical energy takes place by means of a magnetic field. Similar to an electrostatic actuator, there are stationary and moving parts, named the stator and the rotor, respectively. Depending on whether the magnetic field is generated by the static or rotor component, there are two different configurations available for these actuators. Both configurations are described below in detail.

#### 4.3.1. Moving Magnet Configuration

In this configuration of MEMS electromagnetic actuators, a bulk magnet is placed inside an electric coil. When the current flows inside the wire coil, it generates a magnetic field. The intensity of the generated magnetic field depends upon the current passing through the coil, the radius of the coil surface, and the distance from the coil. For a circular coil, the generated magnetic field is given by the Biot–Savart equation:

(28) $$H\left(z\right)=\frac{\mu_{0}NI\;r^{2}}{2{\left(r^{2}+z^{2}\right)}^{3/2}}$$

where *µ*_0_ is the permeability constant, *N* is the number of turns, *I* is the current, *r* is the mean radius, and *z* is the distance along central axis [130].

In the moving magnet configuration, a permanent magnet with net magnetization vector ***M*** is placed inside an external magnetic field ***H****_ext_* created by one or more electric coils at angle *α*. The external field applies a torque on the moveable magnet given by:

(29) $$T_{H}=\left|V_{mag}M\times H_{ext}\right|=V_{mag}MH_{ext}sin\left(\alpha \right)$$

with *V_mag_* being the magnetic volume. Using a soft magnetic material, the generated torque ***T****_H_* rotates ***M*** moving it away from the equilibrium position (easy axis) by an angle *θ*. An anisotropy magnetic torque ***T****_a_* will be generated inside the magnet, tending to realign it to its initial position.

(30) $$T_{a}=-K_{a}sin\left(2\theta \right)$$

*K_a_* is the magnetic-anisotropy constant. An opposite torque ***T****_a_* is exerted on the easy axis and thus on the magnet itself [64]. If the direction of ***H****_ext_* remains constant at an angle *γ* (*α* = *γ* at the beginning) from the easy axis, the torque becomes:

(31) $$T_{H}=V_{mag}MH_{ext}sin\left(\gamma -\theta \right)$$

In a permanent magnet, *M* = *M_s_* (saturated magnetization), and *θ* = 0. The magnetization of soft magnets changes with the applied external magnetic field and *M_s_*.

The external magnetization vector acting along the direction of ***M*** is:

(32) $$H_{a}=H_{ext}cos\left(\gamma -\theta \right)$$

Thus, the change in ***M*** induces the poles at the end of magnet, which generates a demagnetized field ***H****_d_* in the opposite direction of ***H****_a_*:

(33) $$H_{d}=-N_{M}M/\mu_{0}$$

where *N_M_* is the shape anisotropy coefficient. The net field (***H****_i_*) inside the sample changes to the sum of the applied and demagnetized field. The sample moves to reduce ***H****_i_* and cause the magnetization vector to be:

(34) $$M=\min\left[\frac{\mu_{0}H_{ext}\cos\left(\gamma -\theta \right)}{N_{M}},M_{s}\right]$$

In equilibrium, the field torque ***T****_H_* rotates ***M*** from easy axis and is balanced by the anisotropy torque ***T****_a_* and tends to align M and vibrate the magnetic component [131].

**Figure 17:**
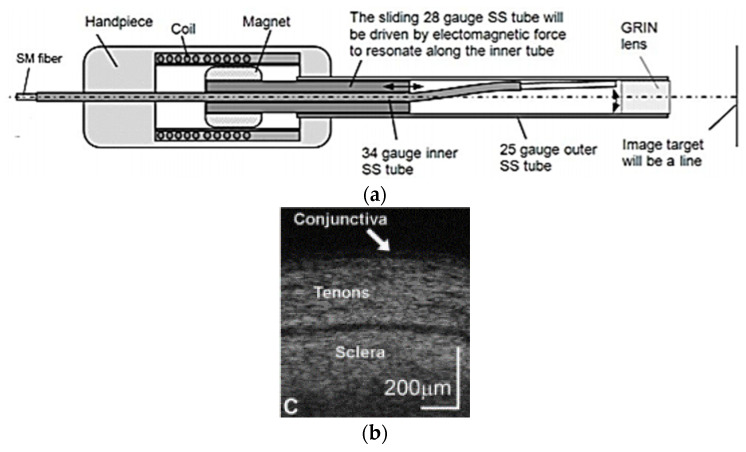
Forward-viewing OCT probe based on electromagnetic actuation: (**a**) schematic diagram; (**b**) real-time image of conjunctiva (taken with the permission of [132]) © The Optical Society.

Joos et al. developed an OCT probe for imaging based on this technique. In this probe, an electromagnetic coil was placed at the outer surface in the center, in which a magnet was placed carrying a thin-walled 28-gauge tube. An SMF fiber was contained in a “S”-shaped 34-gauge stainless-steel tube placed within the 28-gauge tube, as in the schematic shown in Figure 17a. In the presence of an electric current at the coil, the electromagnetic force generated the sliding motion of the 28-gauge tube along the curved part of the inner S-shaped tube, allowing the fiber to move in the lateral direction [132]. The performance of the device was tested by imaging the ocular tissue structures. A real-time OCT image of ocular conjunctiva is shown in Figure 17b, where different tenons and sclera layers can be clearly identified [132].

**Figure 18:**
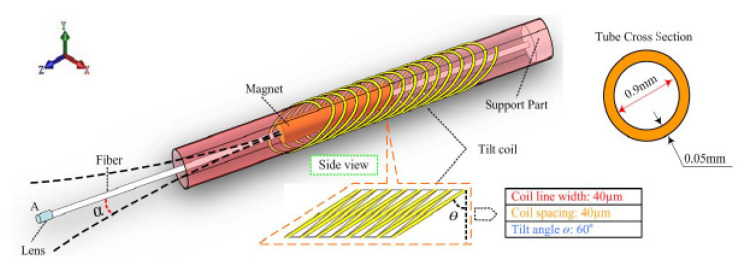
Schematic of an electromagnetically driven fiber scanner (taken with the permission of [133]).

Sun et al. developed a cantilevered fiber scanner for medical endoscopic applications, where an SMF with a collimating lens was excited at resonance using an electromagnetic actuator working on this principle. The researchers fixed a soft cylindrical magnet to an optical fiber using a 1 mm diameter polyimide pipe, and a tilted coil was fabricated using a microfabrication lithography technique [133]. The schematic of the design is illustrated in Figure 18. In the presence of an AC current applied to the coil, a magnetic field was generated within the coil and vibrated the magnet fixed to the fiber, resulting in excitation of the fiber [133]. Two tilted coils can be used to drive the fiber in two directions to obtain a 2D image [134].

**Figure 19:**
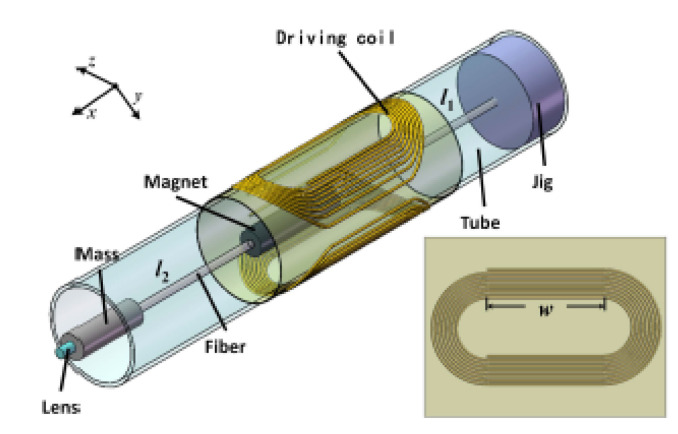
Schematic design of a fiber scanner excited at second resonance mode using an electromagnetic actuator (taken with the permission of [64]).

A similar probe, schematized in Figure 19, was recently developed by Yao et al., where a cantilevered fiber containing a mass element and a lens at its tip was excited at a second resonance mode using a pair of flexible driving coils. The geometry of the cantilevered portion generated a 2D elliptical motion, with a larger scan angle at the fiber tip in the presence of a magnetic force generated by the soft magnet in the presence of a magnetic field [64].

#### 4.3.2. Moving Coil Configuration

In the moving coil configuration, an electric coil is fabricated on the scanner and is placed inside a static magnetic field created by external magnets. When the current flows through the coil in the presence of an external magnetic field, a force is exerted on the coil, designated as the Lorentz force. The force generated on the coil is given by:

(35) F=|IL×B|=BILsin(θ)

where *B* is the external magnetic field, *I* is the current, *L* is the length of the conductor, and *θ* is the angle between the direction of the current and magnetic field [135]. The force produced can be written in terms of Equation (28) as well. Usually, the conductor is placed perpendicularly to the magnetic field to obtain the maximum exerted force. Equation (35) will be simplified to:

(36) F=BIL

In the case of a coil with *N* turns, the generated magnetic torque on the coil is:

(37) $$T_{mag}=2\overset{N}{\underset{n=1}{\sum }}BILr_{n}$$

with *r_n_* being the distance of the nth coil turn from the center [90].

As in the previous case, the actuator (coil surface) deforms due to the generated torque, and a restorative torque will arise in the coil to bring it to its initial state, causing the vibration of the moving coil. This technique is frequently used to actuate micromirror surfaces [136,137] and finds limited use in cantilevered fiber scanners.

#### 4.3.3. Magnetostrictive Actuation

Magnetic materials are characterized by a special property which allows them to change their dimensions in the presence of a magnetic field. This effect is called magnetostriction. The material can undergo a change in dimension until it reaches the value of saturation magnetostriction, which depends on the magnetization and, therefore, on the applied magnetic field [138].

Bourouina et al. developed a 2D optical scanner based on the magnetostrictive effect. In this case, a silicon cantilever was coated with a magnetostrictive film. Due to the uniaxial nature of the magnetostrictive material, bending and torsion vibrations were generated simultaneously in the presence of an AC magnetic field generated by the electric coils placed in its surroundings. Later, a piezoresistive detector was incorporated in the device to measure the bending and torsional vibrations [139,140].

**Figure 20:**
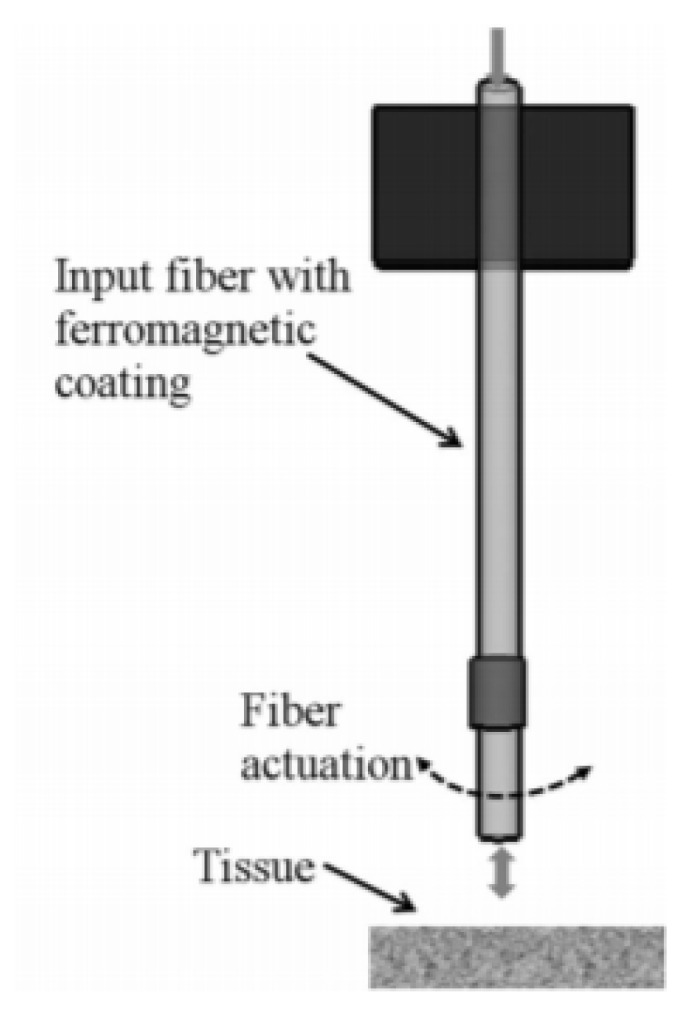
Schematic of a magnetically actuated fiber-based imaging system (reprinted with permission from [141]).

A slightly different fiber optic scanner was developed by a group of researchers from the University of Texas. In this design, an optical fiber was coated with a ferromagnetic gel which experienced a bending motion in the presence of an external magnetic field generated by a magnet placed at the outer surface. The schematic configuration of this device is shown in Figure 20 [141].

### 4.4. Electrostatic Actuators

An electrostatic actuator includes at least two pairs of electrodes attached to two plates separated by a gap. One of these plates is fixed by anchors and is named the stator, while the other plate is able to move and is designated the shuttle. In the presence of a voltage difference between the two plates, an attractive electrostatic force generates among them, causing the movement of the shuttle plate towards the stator. The amount of the electrostatic force generated between the two components depends on the gap and the dielectric constant of the media separating the two plates. The generated electrostatic force is given by:

(38) $$F_{es}=\frac{A\varepsilon V^{4}}{2g^{2}}$$

where *A* is the electrode area, *ε* is the dielectric constant of the air, *V* is the total voltage difference applied to the plates, and g is the air-gap distance [142]. The maximum voltage that can be applied to a pair of electrostatic electrodes is delimited by the pull-in voltage. The electrostatic force increases with the applied voltage until the point when the force causes the two plates to collapse together. The maximum applicable voltage without causing this phenomenon is called pull-in point voltage. The electrostatic actuators can be classified into parallel plate and comb drive, which are described below.

#### 4.4.1. Parallel Plate Actuator

In a parallel plate configuration, two electrodes are placed parallel to each other in an interdigitated finger configuration. In optical scanners, the moving electrode is mostly represented by a polysilicon mirror used to deflect the light.

Another variation of a parallel plate actuator is a system where the moving electrode has a rotational degree of freedom. The application of the voltage to the electrodes in this case causes the rotation of the moving electrode with a tilt angle obtained from the equilibrium between the electrostatic torque generated by the electrostatic force and the restoring torque. The large deflection angle in this case requires a large air gap between the electrodes. The maximum deflection of the rotating electrode should be less than one third of the air gap to avoid the pull-in phenomenon [143].

Most of the torsional electrostatic actuators are divided into small-sized scanner arrays causing large tilting angles with small air gaps. There are some systems developed with tapered electrodes to allow large tilting angles.

One of the main drawbacks of electrostatic actuators is that a large driving voltage is required to obtain moderate deflection angles. It is possible to partially overcome this by using tapered electrodes instead of the parallel-shaped ones [144].

#### 4.4.2. Comb Drive Actuator

In electrostatic comb actuators, multiple plates are connected to make interdigitated static and mobile rows. Such a configuration enables an increase in the interaction area between the two electrodes, and, consequently, high electrostatic forces are generated. As in the parallel plate configuration, the out-of-plane motion of the mobile mirror structure can be obtained by making a vertical offset between the torsional support and the driving arm [143].

In vertical comb drives, the moving comb motion is out-of-plane with the motion of the fixed comb, which avoids the pull-in phenomenon. Moreover, the deflecting mirror can be decoupled from the actuating part, permitting a large possible deflection of the mirror itself. The higher electrostatic torque generated by the comb structure leads to the possibility of higher driving frequencies and thus a higher scan speed [145].

Vertical comb drives are used frequently to actuate micromirrors [146,147,148]. It is possible to place the moving comb structures at a certain angle with respect to the fixed ones to obtain an angular vertical comb drive. The initial angle between the comb structures determines the obtainable maximum angle rotation of the mirror connected to it [61].

A group of researchers at Fraunhofer University studied the design optimization for comb drive micro-actuators. It was more convenient to place the electrodes in a star-shaped pattern to obtain a higher deflection of the mirror surface [149].

**Figure 21:**
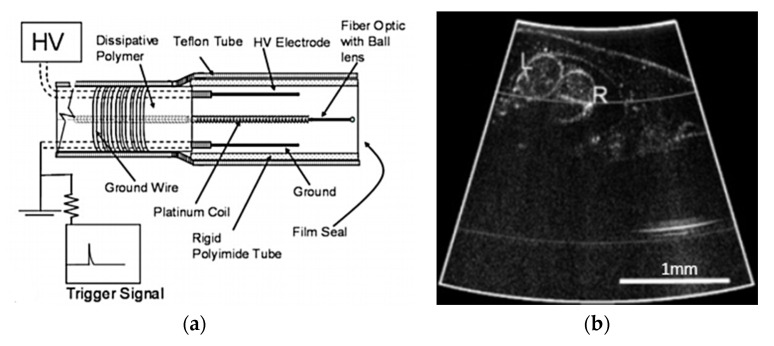
Electrostatically driven fiber scanner: (**a**) schematic diagram; (**b**) Doppler OCT image of a tadpole heart (taken with permission of [150]) © The Optical Society.

Both types of actuators are mainly used to actuate micromirrors [100]. Munce et al. developed an electrostatically driven fiber optic scanner (shown in Figure 21a) where a single-mode fiber was placed in a platinum coil. The packaged probe had a diameter of 2.2 mm. There were two insulated wires placed around the optical fiber which acted as electrodes. An electrostatic force was generated around the fiber in the presence of a potential difference between the two electrodes, which allowed the fiber tip to vibrate [150]. An in-vivo Doppler OCT image of the heart of a Xenopus laevis tadpole, taken with this device (Figure 21b), enabled clear visualization of the left and right aortic arches [150].

### 4.5. Shape Memory Alloy Actuators

Shape memory alloys (SMAs) are unique metallic alloys having the ability to return to their original shape after being deformed plastically. The deformation recovery is usually obtained by increasing the temperature of the material, which releases the state of stress.

SMA material is available in either a wire or a sheet shape. Frequently used SMA actuators take the form of a coil structure as they can provide a larger stroke as compared to a straight wire per unit length. The shear modulus and the spring constant of a SMA coil/spring depend on the composition, temperature, strain, and shape memory treatment applied to the material. In the design of an SMA actuator, the material is deformed at a low temperature and thermally treated to remember its shape. The shape recovery of the coil is obtained by increasing the temperature via the Joule effect by passing a current through the wire [151].

SMA coil actuators show a one-way shape memory effect. Thus, a bias spring or a second SMA coil spring is combined with an SMA coil actuator to obtain a two-way actuation. When a current is passed through the SMA coil, it tends to return to its original shape, which exerts a force on the second spring/coil, permitting motion in one direction. When the current is stopped, the SMA coil cools down and it is re-deformed by the force applied by a bias spring or activating the second coil [152]. 

**Figure 22:**
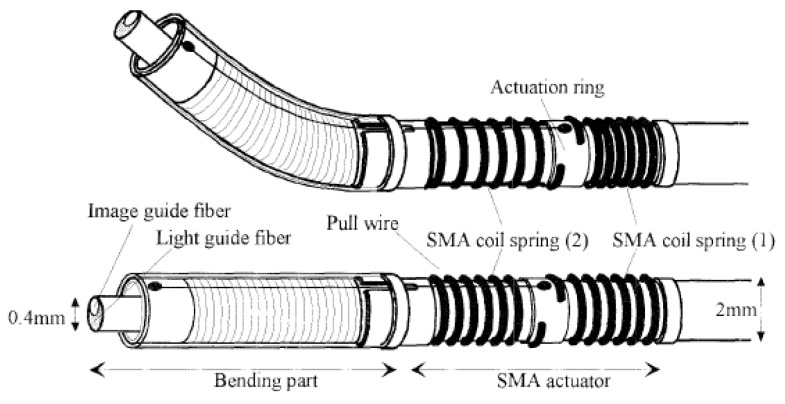
Schematic design of endoscopic tip guided using SMA coils (taken with the permission of [153]).

SMA coils are largely used in endoscopes to bend the distal tip for a long time. Maeda et al. fabricated a 2 mm diameter endoscope head where an actuation ring connected to two SMA springs guided the bending motion of the endoscope tip which contained the optical guide fibers by pulling or releasing the pull wire connected to it. The schematic diagram showing the structure of the described design is shown in Figure 22 [153]. When an SMA coil spring (1) is heated, it tends to recover its shape and rotates the actuation ring to the right, which in turn pulls the wire, causing the bending motion of the tip. By stopping the current in that coil and heating the other coil, the tip returns to its original position [153].

Haga et al. used three SMA coil actuators at equilateral triangle locations between two links to form a joint. The bending motion generated by these actuators allowed the snake-like movement of the central working channel of the endoscope, i.e., guided the endoscope [154].

**Figure 23:**
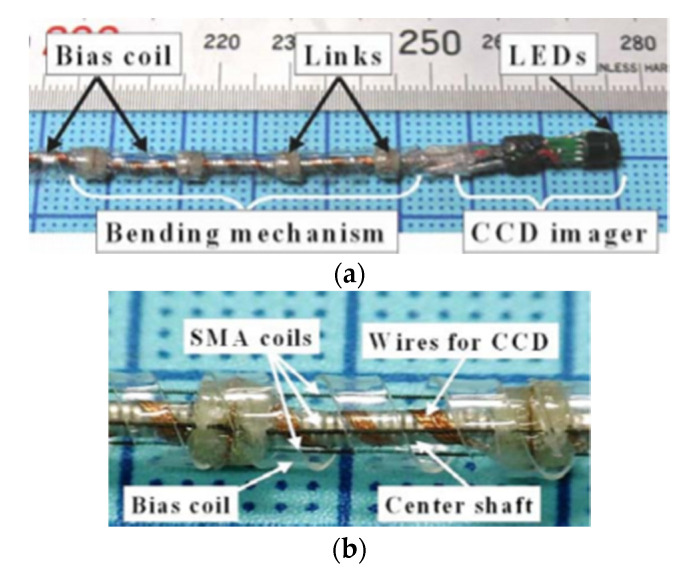
Active bending endoscope using SMA coil actuators: (**a**) endoscope design; (**b**) enlarged view of the actuation mechanism (taken with the permission of [155]).

A similar endoscope was designed by Makishi et al., where the active bending motion of the endoscopic tip, containing a CCD imager, was obtained using three SMA coil actuators [155]. The endoscope design showing the structure of the device is illustrated in Figure 23 [155]. Another similar endoscope was developed by Kobayashi et al., where the bending motion of the endoscopic central channel containing a CMOS imager and three LEDs was obtained using three SMA wires and a stopper coil. In this case, a large bending angle was obtained at low cost by allowing the SMA wires to follow an arc-shaped deformation [71].

## 5. Discussion

Optical endoscopic imaging enabled the visualization of cellular and subcellular structures in real time, enabling early interventions and improved diagnostic yield from fewer biopsies. Moreover, the less invasive nature of the imaging device results in reduced tissue trauma, low risk of complications and operational costs, and fast recovery times. In cantilever fiber endoscopes, an optical fiber is rigidly fixed to an actuator’s substrate surface, leaving a few millimeters of free end at its distal end. Most often, this free end is vibrated at resonance to obtain a large tip displacement, which in these imaging devices is directly related to the resolution of the obtained image. Various imaging applications, such as confocal endo-microscopy, OCT, photoacoustic imaging, etc., can be found using such endoscopes.

Frequently used MEMS actuators in fiber optic endoscopic devices comprise piezoelectric, electrothermal, electromagnetic, electrostatic, and shape memory alloy actuators. Piezoelectric actuators are available in plate or tubular structure, among which the latter is largely used as it provides the base excitation to the fiber held in the center along two directions. By linearly increasing the amplitude of sinusoidal voltages at two pairs of electrodes with a 180° phase shift, one can obtain a spiral scanning pattern.

Electrothermal actuators are available in three different types including hot-and-cold arm, chevron, and bimorph actuators. In a hot-and-cold arm actuator, the different geometries of the two legs produce asymmetric heat generation and, consequently, an asymmetric thermal expansion, generating a bending motion at the actuator tip. Similarly, in bimorph actuators, the different material properties cause the bending of the actuator tip. These techniques can be combined with other actuation methods to obtain a 2D Lissajous scanning pattern. Chevron actuators have symmetrical legs and provide linear 1D motion at the tip. 

Electrostatic actuators are available in parallel plate and comb drive configurations. These actuators find limited use in fiberoptic scanners due to their large dimensions but are frequently used in scanners having proximal scanning devices such as mirrors.

The shape recovery property of shape memory alloy materials makes them an excellent alternative for providing a large actuation force in compact dimensions.

Various imaging endoscopic devices using these actuators are described in this paper. The performance of some recently developed fiber optic cantilever-based scanners is summarized and compared with the clinically available endoscopes in Table 3.

As stated earlier, scanning fiber endoscopes were introduced recently in the field. Most of the devices mentioned in this paper find limited use in clinical application but are more in the transition from research to clinical phase. The only commercially available cantilever-based endoscopes for clinical use are those developed by Pentax [73] and Mauna Kea [32] Technologies.

The choice of an endoscopic device depends on the target imaging region, ease of use, and cost of the device. The cost of an endoscopic device comprises fabrication and operating cost. The operating costs mainly consist in decontamination and sterilization, which can be performed at high temperature such as autoclaving or hot air oven or at low temperature using chemical agents. The fabrication cost of an endoscope highly depends on the cost of the laser source, actuation, and detection mechanisms. Among the various actuators studied in this paper, electrothermal actuators are economic ones. However, the high working temperature can limit their usage at a high frequency. Otherwise, piezoelectric tubular actuators are cost-effective in terms of possible bidirectional actuation at high actuation frequency. The development of MEMS actuators enabled batch production of miniaturized actuators, reducing the fabrication costs to great extent and permitting the fabrication of disposable endoscopic scanners.

## 6. Conclusions

The actuation and scanning devices for cantilever-based endoscopic probes are described in this review paper along with their design and working principles. The size of an actuator is an important factor in a cantilevered endoscopic device. The developments in the MEMS field permit the mass production of small-sized actuators, enabling the fabrication of small-sized imaging probes. The endoscopic technology is aiming towards the design of low-cost disposable imaging devices as the reprocessing and sanitation of an endoscopic device is a complex and expensive process. The fabrication of small-sized actuation mechanisms helps to reduce the price of device. Moreover, small-sized probes can be advanced further to image the small body cavities, enabling early detection of pre-cancerous surfaces and helping in the diagnostic procedure to control the lesion at a preliminary stage.

## Figures and Tables

**Table 1:** Comparison between system performances of imaging technologies used in endoscopes.

	Pixel Density (Pixels/mm^2^)	Image Resolution (Pixels)	Pixel Size	Advantages	Disadvantages
CFB	113 k	30.0 k/64.0 k	2 µm Ø	Small form factor, low cost	Cross-coupling and honeycomb effect degrade image resolution Aging effect results in non-working pixels due to fractured fibers within the bundle
CCD	238 k	95.0 k	0.5 µm × 0.5 µm	Small pixel size, low cost, no aging effect	Rectangular geometry limits the usable area, low dynamic range, poor light collection in low illumination area
CMOS	476 k	190 k	1.45 µm × 1.45 µm	Higher image resolution, low cost	Rectangular geometry limits the usable area, poor resolution in devices with diameter < 1 mm
SFE	345 k	282 k	Dependent on scanning pattern and sampling rate	Higher sampling rate and resolution in sub-millimeter-sized devices	Performance dependent on actuation method and sampling rate. Spatial point spread dependent on objective lens at the tip and illumination properties.

**Table 2:** Comparison between different actuation methods used in cantilevered endoscopes [6].

	Electrostatic	Electro-Thermal	Piezoelectric	Electromagnetic	Shape Memory Alloy
Force	**✓**	**✓**	**✓✓✓**	**✓✓**	**✓✓✓**
Displacement amplitude	**✓✓**	**✓✓✓**	**✓**	**✓✓✓**	**✓✓**
Compactness	**✓✓✓**	**✓✓✓**	**✓✓**	**✓**	**✓✓**
Power consumption	**✓✓**	**✓**	**✓**	**✓✓✓**	**✓✓✓**
Working principle	Electrostatic force	Thermal expansion	Piezoelectric effect	Magnetization effect	Material deformation
Motion range	1D/2D	1D/2D	2D	1D	1D
Scanning pattern	Spiral	Lissajous	Spiral	Linear	Linear
Advantages	Fast response, low voltage required, easy fabrication, and no hysteresis	Large displacement, low operating voltage, small dimensions	Large force generated, wide operating frequency range, low power consumption	Large displacement obtained, quick and linear response, easy to control	Flexibility, large frequency response
Disadvantages	Large device dimensions, pull-in problem, complicated circuit	High working temperature, not operable at very high frequencies	Limited displacement	Large device dimensions, difficult to manufacture	Low displacement

**Table 3:** Fiber-optic cantilevered scanners developed for endomicroscopy.

Actuation Principle	Imaging Modality	Scanning Direction	Resolution	FOV	Frequency	Driving Voltage	Frame Rate	Scanner Dimensions	Scanning Pattern	Cantilever Fiber	Refs.
Piezoelectric bimorph	Multiphoton	Forward	0.8 µm (lateral), 10 µm (axial)	110 µm × 110 µm	1.05 kHz (fast axis), 4.1 Hz	50 Vpp (resonant bimorph) 200 Vpp (non-resonant)	4.1 fps (512 × 512 pixels)	3 mm (diameter) 40 mm (rigid length)	Raster scan	DCF	[105]
Piezoelectric tube	Two-photon	Forward	0.61 µm ÷ 1.10 µm (from center of FOV to peripheric zone)	160 µm	3.1 kHz	48 Vpp	6 fps	~2 mm (diameter)	Spiral	DCF with GRIN lens	[156]
Piezoelectric tube	Two-Photon	Forward	10 µm	70°	5 kHz	25 V	15 fps	1.6 mm (diameter)	Spiral	SMF	[16]
Piezoelectric thin film	E-OCT	Side-view	5 µm (axial)	152°	394 Hz	1.3 Vpp	-	3.4 mm × 2.5 mm	radial	-	[157]
Electrothermal	Confocal endomicroscope	Forward	~1.7 µm	378 µm × 439 µm	239 Hz (x-axis) 207 Hz (y-axis)	16 Vpp	1 fps	1.65 mm (diameter) 28 mm (rigid length)	Lissajous	SMF	[65]
Electrothermal	OCT (A-scan)	Forward	~17 µm (lateral) ~9 µm (axial)	~3 mm (beam scanning)	~100 Hz	3 Vac_pp, 1.5 V DC offset	200 fps	5.5 mm (diameter) 55 mm (rigid length)	raster	-	[96]
Electromagnetic	OCT (B-scan)	Forward	4–6 µm (axial) 25–35 µm (lateral)	2 mm	5 Hz	±10 V (triangle wave)	-	0.51 mm (diameter)	Linear	SMF	[132]
Micromotor	PA and US endoscopy	Side-view	~58 µm (PA radial) ~30 µm (US radial) ~100 µm (PA transvers) ~120 µm (US transverse)	~310°	~4 Hz	~3.2V DC	4 fps	2.5 mm (diameter) ~35 mm (Rigid length)	Radial	MMF	[40]
Electromagnetic	Confocal	Forward	0.8 µm (lateral)	390 µm × 390 µm	700 Hz (fast scan) 1–2 Hz (slow scan)	-	1 fps	8 mm (diameter)	Raster	SMF	[158]
Cellvizio	Confocal	Forward	5–15 µm (axial) 2–5 µm (lateral)	600 µm × 500 µm	-	-	12 fps	2.5 mm	-	-	[33]

## Data Availability

No new data were created or analyzed in this study. Data sharing is not applicable to this article.
